# PROSPER: An Integrated Feature-Based Tool for Predicting Protease Substrate Cleavage Sites

**DOI:** 10.1371/journal.pone.0050300

**Published:** 2012-11-29

**Authors:** Jiangning Song, Hao Tan, Andrew J. Perry, Tatsuya Akutsu, Geoffrey I. Webb, James C. Whisstock, Robert N. Pike

**Affiliations:** 1 Department of Biochemistry and Molecular Biology, Monash University, Melbourne, Australia; 2 National Engineering Laboratory for Industrial Enzymes and Key Laboratory of Systems Microbial Biotechnology, Institute of Industrial Biotechnology, Chinese Academy of Sciences, Tianjin, Tianjin, People's Republic of China; 3 Bioinformatics Center, Institute for Chemical Research, Kyoto University, Uji, Kyoto, Japan; 4 Faculty of Information Technology, Monash University, Melbourne, Australia; 5 ARC Centre of Excellence in Structural and Functional Microbial Genomics, Monash University, Melbourne, Australia; Indian Institute of Science, India

## Abstract

The ability to catalytically cleave protein substrates after synthesis is fundamental for all forms of life. Accordingly, site-specific proteolysis is one of the most important post-translational modifications. The key to understanding the physiological role of a protease is to identify its natural substrate(s). Knowledge of the substrate specificity of a protease can dramatically improve our ability to predict its target protein substrates, but this information must be utilized in an effective manner in order to efficiently identify protein substrates by *in silico* approaches. To address this problem, we present PROSPER, an integrated feature-based server for *in silico* identification of protease substrates and their cleavage sites for twenty-four different proteases. PROSPER utilizes established specificity information for these proteases (derived from the MEROPS database) with a machine learning approach to predict protease cleavage sites by using different, but complementary sequence and structure characteristics. Features used by PROSPER include local amino acid sequence profile, predicted secondary structure, solvent accessibility and predicted native disorder. Thus, for proteases with known amino acid specificity, PROSPER provides a convenient, pre-prepared tool for use in identifying protein substrates for the enzymes. Systematic prediction analysis for the twenty-four proteases thus far included in the database revealed that the features we have included in the tool strongly improve performance in terms of cleavage site prediction, as evidenced by their contribution to performance improvement in terms of identifying known cleavage sites in substrates for these enzymes. In comparison with two state-of-the-art prediction tools, PoPS and SitePrediction, PROSPER achieves greater accuracy and coverage. To our knowledge, PROSPER is the first comprehensive server capable of predicting cleavage sites of multiple proteases within a single substrate sequence using machine learning techniques. It is freely available at http://lightning.med.monash.edu.au/PROSPER/.

## Introduction

Proteases, also known as peptidases, proteinases or proteolytic enzymes, are enzymes that hydrolyze amino acids bonds not only in proteins, but also in peptides [1]–[6]. This process is used as a biological switch to activate/deactivate protein function in numerous biological processes. Indeed, controlled proteolysis is a major pathway through which the estimated 1–1.5 million peptides and proteins needed to fulfill the complexity of human life are produced from ∼26,000 human genes. Proteases represent ∼2% of all gene products in humans (about 500–600 proteases), reflecting their diverse functional roles in many biological processes. Proteases thus have central roles in “life and death” processes, such as neural, endocrine and cardiovascular signaling, digestion, degradation of misfolded or unwanted proteins, immunity, cell division and apoptosis. Accordingly, proteases have also been implicated in many disease processes [1]–[3].

The key to understanding the physiological role of a protease is to identify the repertoire of its natural substrate(s) [7], [8]. Proteases act as processing enzymes that carry out either highly or moderately selective cleavage of the scissile bond within the cleavage site of their substrates. Thus, the specificity of proteases varies, primarily depending on their active sites, which display selectivity ranging from preferences for a number of specific amino acids at defined positions, to more generic proteases with limited discrimination at one position. In addition to the primary amino acid sequence of the substrate, the substrate specificity of a protease is also influenced by the three-dimensional conformation of its substrates. In particular, proteases preferentially cleave substrates within extended loop regions, while residues that are buried within the interior of the protein substrate are usually inaccessible to the protease active site. In addition to the sequence and structure determinants, substrate specificity and selectivity can also be influenced by the presence of the so-called exosites that are located outside the active site. Moreover, protease activity is also regulated by co-factors, ligands or other proteins that reversibly bind to proteases in an allosteric manner and finally affect the activity [2], [9], [10]. This is particularly the case for proteases such as the matrix metallopeptidases and thrombin. Through providing additional binding regions not influenced by the primary specificity subsites, exosite interactions can modulate the substrate specificity of the protease. For certain substrates, exosite binding and interaction is an absolute requirement in order for the cleavage to occur. Finally, cleavage is regulated by the temporal and physical co-location of the protease and the substrate. For example, some proteases are sequestered within specific compartments, with limited access to proteins, while others are able to cleave multiple substrates in different physiological compartments [8].

In recent years, high-throughput mass spectrometry techniques or specificity profiling of peptide libraries have typically been used to identify novel cleavage sites in protease substrates [11]–[20]. However, experimental identification of protease cleavage events, in general, is a difficult, labor-intensive and time-consuming task and requires access to specialised equipment. In addition, high-throughput proteomics techniques suffer from some intrinsic limitations. For example, while they tend to provide close-to-complete fractional sequence coverage by detecting isolated proteins or peptides, in most cases, they fail to detect low-abundance proteins that might also be produced by proteolytic events. As a result, the complete repertoire of protease substrates remains to be fully characterized for most enzymes.

In contrast to experimental methods, *in silico* prediction of substrate cleavage sites has emerged as a useful alternative approach to provide valuable insights into complex enzyme-substrate interaction relationships. Efficient computational tools would reduce the number of experiments to be performed to identify physiologically relevant substrates. A number of computational methods have been developed to predict substrate cleavage sites for proteases. They can be broadly classified into two types: machine learning-based or empirical scoring function-based.

The first group applies machine learning algorithms to train models from a training set of peptides with known cleavage site information. These methods are based on selection and representation of useful features and training of predictive models from the given samples. Various types of features and machine learning methods have been explored [21]–[28]. These methods usually take known substrate peptide sequences as the input to machine learning models and the trained models can predict cleavage sites with accuracies from 70% to 90%, based on different training datasets. The second group of methods identify substrate cleavage sites by learning the underlying rules based on the distribution of positive and negative samples and building empirical scoring functions to discriminate between the two classes. Tools falling in this category include PeptideCutter [29], CasPredictor [30], GraBCas [31], PoPS [32] and SitePrediction [33]. These methods usually either calculate a frequency score for the positions surrounding a potential cleavage site or use a similarity score based on an amino acid substitution matrix in combination with extra features, such as secondary structure and solvent accessibility information, which might help to interpret prediction results (see reference 8 for a comprehensive review).

Despite this recent progress in developing *in silico* prediction tools for protease cleavage sites, they have certain limitations, principally their prediction performance, which varies considerably. A major underlying reason is the use of several different training datasets of varying quality and size, but with high-quality and high-throughput proteome-wide profiling data being deposited in comprehensive databases [4], [5], [34], [35], it is now imperative and necessary that benchmark training and test datasets with high quality be curated by taking full advantage of these resources. A second issue is that only PeptideCutter [29], PoPS [32] and SitePrediction [33] were implemented to model and predict substrate cleavage sites for more than one protease family. For instance, CasPredictor [30], GraBCas [31] and Cascleave [28] can only be used to predict cleavage sites of caspases/granzyme B, but it is not feasible to apply them to predict cleavage sites of other proteases. The third issue is how to characterize efficient and useful features that better describe the properties of protease cleavage sites and contribute to performance improvement. Recent work suggested that it was useful to include local sequence environment surrounding potential cleavage sites and additional features such as predicted structural information in the form of secondary structure, solvent accessibility and native disorder [27], [28], to improve the prediction of cleavage sites of caspases, but the overall contribution of these features needs to be examined and validated across more protease families. In addition, there is a need to address the highly imbalanced nature of protease specificity data (cleavage sites are greatly outnumbered by sites that are not cleaved) and how to filter out false positives. These two issues have particularly important ramifications for proteome-wide predictions, because only high-confidence predictions are of interest.

To address the limitations of existing tools and to improve the performance of protease substrate cleavage site prediction, here we have developed a new bioinformatics tool- PROSPER (PROtease substrate SPecificity servER). We addressed the problem of predicting substrate cleavage sites for different protease families based on the amino acid sequences of substrates, by formulating the cleavage site prediction problem as a binary classification task and solving it with sophisticated machine learning techniques. High-quality large training datasets were curated by taking advantage of the experimentally verified substrate cleavage sites of various protease types in the MEROPS database [34], [35]. The curated datasets covered the four major catalytic types (aspartic, cysteine, metallo and serine) and consisted of 24 different protease types with varying substrate specificity profiles. PROSPER is an integrated multiple feature-based tool, which we used to extensively examine the influence of several different sequence encoding schemes based on different combinations of features on the prediction performance of the PROSPER models. These results indicate that PROSPER provides superior prediction performance in comparison with other tools. PROSPER was used to generate high-stringency predictions of putative cleavage sites for caspases and granzyme B enzymes, which might be useful in identifying physiologically relevant substrates for these enzymes. Taken together, PROSPER is anticipated to be a useful tool for *in silico* identification of cleavage sites of proteases within physiological substrates.

## Materials and Methods

### Data collection

#### Non-redundant Dataset Construction

We used the MEROPS database [34], [35] as a comprehensive database for proteases and their substrates and extracted protease-specific substrate sequences and their cleavage sites. We also cross-referenced the CutDB [4] and PMAP [5] databases. All of the substrate cleavage sites were experimentally verified. For the sake of efficient construction of machine learning models, only proteases having at least 40 experimentally verified substrates at the time of inception of the study were considered. In addition, exopeptidases (aminopeptidases, carboxypeptidases, etc) and oligopeptidases were generally not included in this study. Moreover, because we are interested in predicting cleavages within native proteins, peptidases that work at pH extremes and are likely to degrade only denatured proteins were also excluded. The issue of selection bias in the curated datasets was addressed by performing sequence homology reduction: the CD-HIT algorithm [36] was used with a threshold of 70% sequence identity to cluster homologous sequences in the current dataset. This step is necessary to eliminate sequence redundancy and avoid overestimation of the prediction performance of machine learning models.

After sequence homology reduction, the final dataset contains 24 proteases, 3520 substrate sequences and 5635 cleavage sites, covering the four major catalytic types- Aspartic (A), Cysteine (C), Metallo (M) and Serine (S). Table 1 lists the number, type and the P4-P4′ cleavage pattern of these proteases as described by MEROPS. The complete list of substrate sequences and cleavage sites for each protease can be found at http://lightning.med.monash.edu.au/PROSPER/.

**Table 1:** Summary of the number, type and the P4-P4′ cleavage pattern of protease substrates as described by the MEROPS database.

Protease family	Protease	Merops ID	Number of substrate sequences	Number of cleavage sites	Cleavage pattern (P4-P4′) in MEROPS
**Aspartic protease**	HIV-1 retropepsin	A02.001	239	376	-/-/VE/L†L/EVA/-/-
**Cysteine protease**	Cathepsin K	C01.036	69	85	-/-/LPV/EA†GE/-/-/-
	Calpain-1	C02.001	42	82	-/-/L/-†AS/-/-/-
	Caspase-1	C14.001	41	50	DL/EV/-/D†SG/-/-/-
	Caspase-3	C14.003	235	347	D/E/V/D†GS/-/-/-
	Caspase-7	C14.004	74	89	DES/E/V/D†G/-/-/-
	Caspase-6	C14.005	62	168	V/ED/-/D†-/-/-/-
	Caspase-8	C14.009	41	58	DL/ES/-/D†GS/-/-/-
**Metalloprotease**	Matrix metallopeptidase-2	M10.003	575	1185	-/P/-/-†LI/-/-/-
	Matrix metallopeptidase-9	M10.004	44	211	G/PA/-/G†L/-/GA/-
	Matrix metallopeptidase-3	M10.005	53	152	-/PA/A/-†L/-/-/-
	Matrix metallopeptidase-7	M10.008	43	95	-/PAG/-/G†L/-/-G/-
**Serine protease**	Chymotrypsin A (bovine)	S01.001	161	293	-/-/-/YFL†-/-/-/-
	Granzyme B (human)	S01.010	235	276	V/-/-/D†-/-/-/-
	Elastase-2	S01.131	154	249	-/-/-/VIAT†-/-/-/-
	Cathepsin G	S01.133	121	176	-/V/L/LHF†S/-S/A/V
	Granzyme B (mouse)	S01.136	148	154	V/-/-/D†-/-/-/-
	Thrombin	S01.217	76	94	-/-/PLAG/R†SAG/-/-/-
	Plasmin	S01.233	44	96	-/-/-/KR†-/-/-/-
	Glutamyl peptidase I	S01.269	377	703	-/-/-/E†-/-/-/-
	Furin	S08.071	72	82	R/-/KR/R†S/-/-/-
	Signal peptidase I	S26.001	269	269	-/A/-/A†A/E/-/-
	Thylakoidal processing peptidase	S26.008	42	43	PS/A/-/A†-/E/-/-
	Signalase	S26.010	303	303	-/AVS/-/A†A/-/-/-

“†” indicates the substrate cleavage site after the P1 position.

Positive (cleavage site) and negative (non-cleavage site) peptide sequences of each protease were generated and used as training data. A sliding window strategy was commonly employed to extract local sequence features from both positive and negative data, in which the P1 cleavage site is either symmetrically or non-symmetrically flanked by upstream and downstream residues. As described previously [28], peptide sequences in the positive and negative datasets were extracted using a local sliding window surrounding experimentally verified cleavage sites and other sites that were not cleaved by the corresponding protease. Since previous work indicated that predictive models based on a local window of P4-P2′ sites achieved the best overall performance [28], in this study, the sequence-based features were also derived using this fixed local window size in order to examine the influences of sequence-based features on the predictive performances of the PROSPER models. In addition, at the feature selection stage, we extended the local window size to P8-P8′ to perform extensive feature selection to extract more relevant features (Table S6).

The number of negative samples is much larger than that of positive ones (thousands of non-cleavage sites versus 5635 cleavage sites), leading to a class imbalance problem and biased model training in favor of negative samples. This issue can be addressed by either increasing the size of the under-represented class by random resampling of the original dataset or decreasing the size of the over-represented class by random resampling of its samples [37], [38]. We adopted the second strategy to overcome the imbalance issue by setting the ratio of the positive to negative samples at 1∶3, as previously suggested [28], [39].

#### Sequence-derived feature extraction

**Figure 1:**
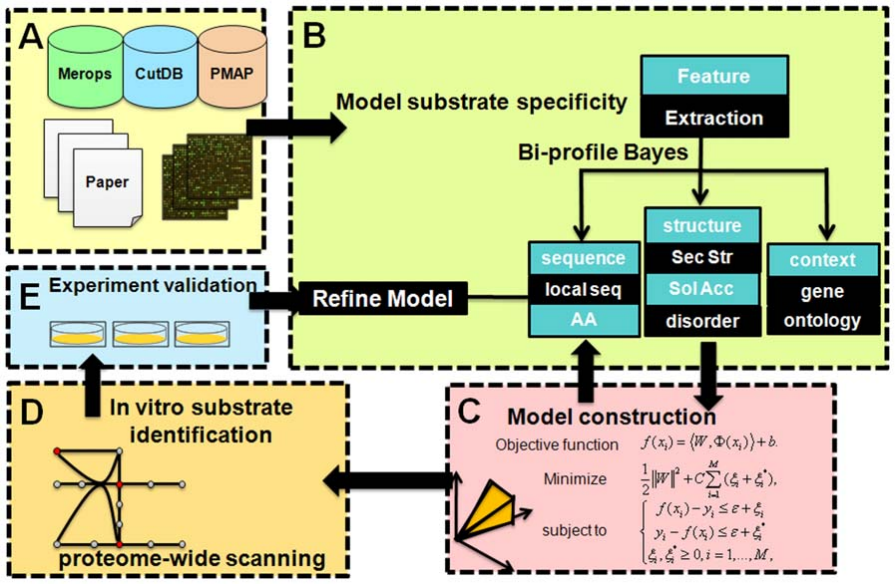
Schematic overview of the PROSPER approach. There are several stages: (A) training datasets and independent test dataset of protease substrates were extracted from multiple resources. These included major comprehensive databases such as MEROPS, CutDB and PMAP, as well as recent proteome-wide profiling studies or the literature. (B) Useful sequence and structure features flanking the cleavage sites were derived and investigated, including local amino acid sequences, predicted secondary structure, solvent accessibility and native disorder. (C) The derived sequence and structural features were entered, following which cleavage probability models were built based on support vector regression (SVR) from the training dataset. In particular, the bi-profile Bayesian feature extraction was applied to extract and integrate the derived features into SVR models, which have been shown to be able to further improve prediction performance. (D) After building the PROSPER models, substrate sequence scanning predictions were made, and (E) PROSPER was further validated using a set of recently identified novel substrates reported in the literature or experimentally verified using positional proteomic approaches.

A schematic overview of our PROSPER approach is illustrated in Figure 1. Input features used by PROSPER are briefly described below.

#### Binary encoding amino acid sequence (BEAA) profiles

At the sequence level, sequence information is encoded using binary encoding amino acid (BEAA) profiles, as previously described [22], [23], [26], [28]. Local amino acid sequences that consist of a fixed number of amino acids on both sides of cleavage sites were extracted and transformed into (*L*×20)-dimensional vectors using an orthonormal encoding scheme, where *L* is the local window size defined as the number of residues involved in the local sequence segment surrounding the potential cleavage site, and each amino acid is represented by a 20-dimensional binary vector with one element set to one and the rest to zero. Local window sizes of *L* = 6 (i.e. P4-P2′) and *L* = 16 (i.e. P8-P8′) were used to train and build the PROSPER models. The latter is considered to include more informative features in feature selection process.

#### Predicted structural features

In addition to local sequence information, local structural determinants were taken into account in the PROSPER models in the form of predicted secondary structure, solvent accessibility and natively unstructured regions.

#### Secondary structure features

Although proteases are generally thought to cleave solvent exposed, flexible, less structured and disordered regions [40], analysis of caspase substrates revealed a considerable proportion of the cleavage sites located in α-helices and β-strands [7], [14], [27], [28]. We predicted the three-state (α-helix, β-strand and other) secondary structure probabilities using PSIPRED [41], which were input to PROSPER models using a local window size of *L*. It has been shown that PSIPRED-predicted secondary structure is useful for improving the performance [42]–[49].

### Solvent accessibility features

Appropriate surface presentation of cleavage sites in a solvent exposed region is particularly important for efficient proteolysis [8], [27], [50]. We thus predicted the two-state solvent accessibility for each residue using ACCpro in the SCRATCH package [51], which provides the estimated probability of a residue being solvent exposed (E) or buried (B) within the substrate structure. Incorporation of this feature has been shown to improve the performance [44]–[46], [52]–[54].

### Native disorder features

Native disorder profiles for potential cleavage sites (or non-cleavage sites) were extracted from the output of DISOPRED2 [55], which provides the predicted probability of a residue being disordered (denoted by “*”) or ordered (denoted by “.”) within the substrate, given a local window size of *L*. The extracted disorder probability matrices were taken as inputs into PROSPER models.

### Cleavage scoring of potential cleavage sites by a machine learning approach

Substrate cleavage site prediction can be formulated as a binary classification problem, i.e. being classified as either a cleavage or non-cleavage site. Here, we employed a machine learning technique, support vector machine (SVM), to solve the difficult task of predicting substrate cleavage sites of different proteases. SVM is an efficient classification algorithm suitable for solving binary classification or multiple classification problems. Based on structural risk minimization from statistical learning theory [56], SVM is able to distinguish positive from negative samples by transforming the data into a higher dimensional space and constructing an optimal separating hyperplane by the use of kernel functions, where two linearly non-separable classes of samples can become separable [57]. We used the support vector regression (SVR) mode in SVM to make a quantitative prediction of the cleavage probability scores for potential cleavage sites of proteases from substrate sequences. The real-value probability score generated by SVR represents the confidence of the prediction, which is very useful and informative. Due to its excellent regression ability, SVR has attracted recent interest with a growing number of applications in the fields of bioinformatics and computational biology [58]–[60].

The SVM_light software [56] was used as the SVR implementation. SVR classifiers were trained using the Radial Basis Function (RBF) Kernel. In the RBF kernel, two important parameters *C* and γ need to be adjusted: *C*, also called cost factor, is a regularization parameter that controls the trade-off between maximizing the margin and minimizing the prediction error, while γ is a kernel-type parameter that dominates the generalization ability of SVR by regulating the amplitude of the kernel function. For each type of protease, we optimized the training parameters of SVR based on 5-cross-validation tests, using a ratio of positive to negative samples of 1∶3 to build the models. In the final cleavage site prediction, a peptide sequence with a predicted cleavage score larger than a given threshold was accepted as cleavage, while those with predicted cleavage scores lower than the given threshold were predicted to be non-cleavage sites. However, the settings of this threshold varied according to the protease type to obtain the best predictive performance, which is subject to the balance of Specificity and Sensitivity values. We could predict cleavage sites with reasonable confidence at appropriate Sensitivity and Specificity levels by controlling the prediction stringency at proper thresholds.

### Sequence encoding scheme

The derived features were encoded into SVR models using bi-profile Bayesian feature extraction [28], [39]. In our previous work, we showed that bi-profile Bayesian feature extraction was useful for improving performance [28]. In this study, a sliding window technique was used to extract and encode features surrounding the cleavage sites using the bi-profile Bayesian feature extraction approach. In addition to the binary encoding amino acid (BEAA) profile, features extracted could be divided into four different types: (i) bi-profile Bayesian amino acid profile (BPBAA); (ii) bi-profile Bayesian secondary structure profile (BPBSS); (iii) bi-profile Bayesian solvent accessibility profile (BPBSA); and (iv) bi-profile Bayesian disordered profile (BPBDISO). Given a potential cleavage site, its feature vector for entry into the model will be encoded by concatenating the constitutive features of the corresponding scheme. For example, in the case of the encoding scheme “BEAA+BPBAA+BPBSS+BPBSA+BPBDISO” (also called “ALL” because it combines all features) and a local window size of *L*, the residues will be represented in a feature vector with (*L*×20+*L*×2+*L*×2+*L*×2+*L*×2 = 28*L*) elements.

### Feature selection

We further selected the optimal features from a total feature set of 448 using an extended local window of P8-P8′, which was based on the sequence encoding scheme “ALL” and which includes all the relevant sequence and structure features. The importance of various features in the set is measured using the mean decrease Gini index (MDGI) by the random forest (RF) algorithm (implemented by the R random forest package) [61]. The MDGI score represents the importance and contribution of an individual element in the feature vector for correctly classifying a residue into a cleavage site or non-cleavage site. To identify more informative features compared to other features in the feature set, a Z-score is calculated for the Gini score of each vector element as:
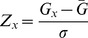
where *G_x_* is the Gini score for the *x*-th feature, 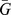 is the average Gini score for all the features in the set and *σ* is the standard deviation. Features with a Z-score greater than a given threshold were considered to be more informative and would be used for training the cleavage site prediction model. Vector elements with a Gini Z-score greater than 1.0 were selected as the optimal features. The MDGI-based feature selection was successfully used by Ebina *et al.* to significantly improve the prediction of protein domain linkers [62]. It is especially attractive for optimal feature selection from a large set with hundreds or thousands of different features.

### Performance evaluation

To objectively evaluate the predictive performance, we performed 5-fold cross-validation, self-consistency and independent tests. In the case of 5-fold cross-validation, substrate sequences in the dataset were randomly divided into 5 equally sized subsets. In each validation step, one subset was reserved as test data, while the remainder were used as training data. This procedure was repeated five times using each subset independently as the evaluation test set. In the case of self-consistency test, substrate sequences in the training set were predicted with a self-trained model. The accuracy of the self-consistency test reveals the fitting ability of the data, reflecting the rigor and consistency of the prediction system. In independent test, the set of cleavage sites used to derive the training model were independent from that used to test the model, with no overlap between the two datasets.

The predictive performance was evaluated using the following measures:

1. Sensitivity (percentage of correctly predicted substrate cleavage sites):
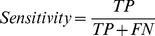

2. Specificity (percentage of correctly predicted non-cleavage sites):
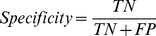

3. Accuracy (percentage of correct predictions for both cleavage and non-cleavage sites):
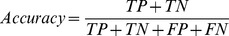

4. Matthew's Correlation Coefficient (MCC), a measure of the quality of binary classifications [63]. MCC = 1 signifies a perfect classification, while MCC = 0 indicates a completely random classification. It is defined as:
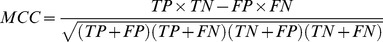

The *F*-score, which is a harmonic mean of precision and recall, is given as:
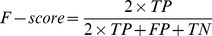
In each of these measures, *TP*, *TN*, *FP* and *FN* denote the number of true positives, true negatives, false positives and false negatives, respectively. The Area under the receiver-operating curve (AUC) was also calculated to compare the performance between different models. We also performed an independent test to compare the performance of PROSPER with other previously developed tools.

## Results and Discussion

### Amino acid preferences in substrate cleavage sites

**Figure 2:**
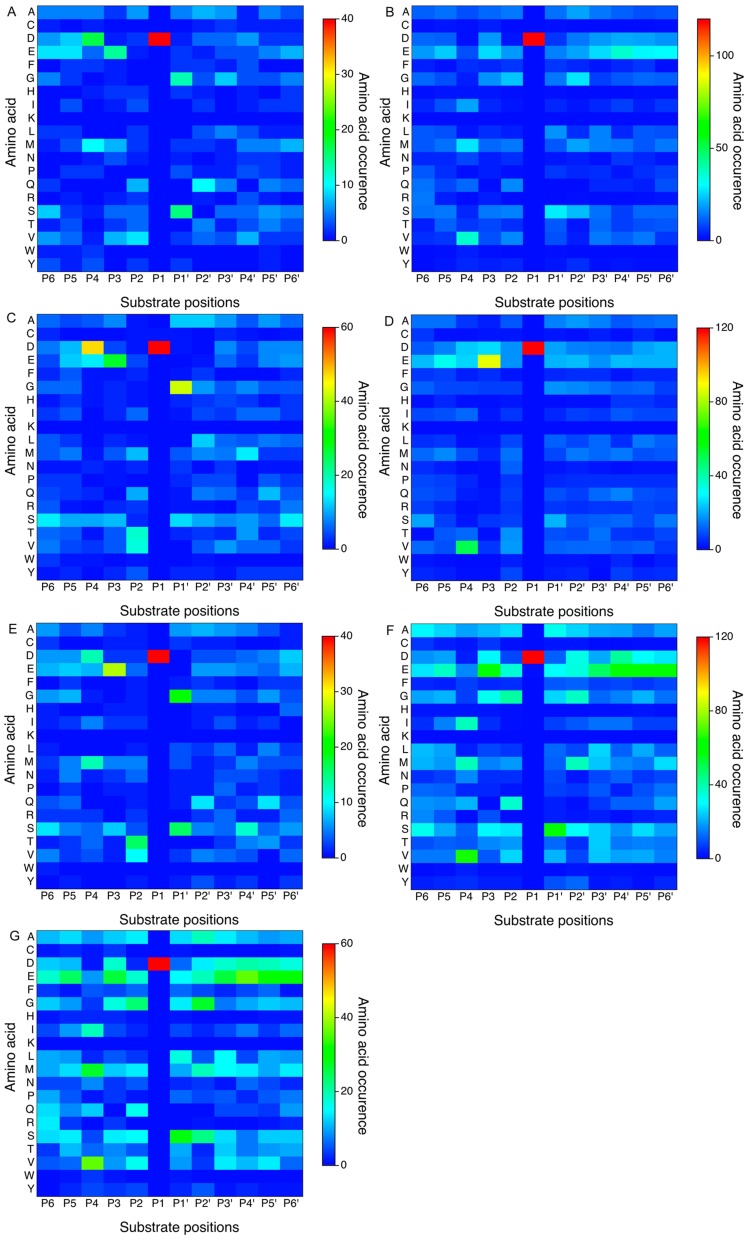
Amino acid occurrences in P6-P6′ positions for the cleavage sites of caspase-1, 3, 7, 6, 8, granzyme B (human) and granzyme B (mouse), displayed in the form of a two-dimensional heat map. Panels A to G correspond to caspase-1, 3, 7, 6, 8, granzyme B (human) and granzyme B (mouse), respectively. Heat map diagrams were rendered using the pro Fit program from QuantumSoft [15].

**Figure 3:**
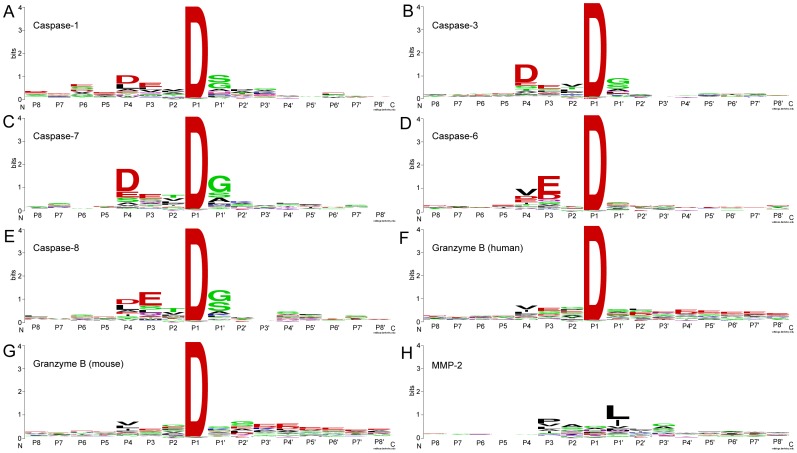
Sequence logo representations of the occurrences of amino acid residues in the cleavage site P8-P8′ positions of caspase-1, 3, 7, 6, 8, granzyme B (human) and granzyme B (mouse). Panels A–G correspond to caspase-1, 3, 7, 6, 8, granzyme B (human) and granzyme B (mouse), respectively. Here, extended window sizes for the P8-P8′ sites were examined in order to cover more specificity determining positions. The sequence logo diagrams were generated using the WebLogo program [96]. To better reflect the occurrence rate of each amino acid type, the sequence logo ordinates have been scaled in bits.

Based on the compiled substrate datasets, we analyzed the statistical distributions in substrate cleavage sites for the twenty-four proteases (Table 1). According to the nomenclature of Schechter and Berger [64], amino acids in the substrate sequence are numbered outward from the cleavage site as …-P4-P3-P2-P1-P1′-P2′-P3′-P4′-…, with the scissile bond located between the P1 and P1′ sites. Taking caspases and granzyme B as an example, the amino acid occurrences in the P6-P6′ positions for the cleavage sites of caspase-1, 3, 7, 6, 8, granzyme B (human) and granzyme B (mouse), were calculated to generate heat map and sequence logo diagrams, which were helpful to identify conserved and frequently occurring amino acids at positions flanking the cleavage site (Figure 2 and 3, respectively). More results regarding other proteases may be found in Figure S1 and the online webpage of PROSPER (http://lightning.med.monash.edu.au/PROSPER/downloads.html).

In general, stronger amino acid preferences were noted on the non-prime side (especially P1 to P4 positions) of the cleavage sites; in contrast, less selectivity was observed on the prime side, except for the P1′ position. As expected, one of the hallmarks of the substrate specificities of caspases is that they preferentially cleave after Asp residues at both P1 and P4 positions (Figure 2), forming the well-known canonical DXXD motif [65], [66]. This applies to all of the caspases, including caspase-1, 3, 7, 6 and 8. According to our analysis, depending on the caspase, 99.7–100% of caspase substrates have a P1 Asp residue, and 14–53% of caspase substrates have a P4 Asp residue. The serine protease, granzyme B (both human and mouse), shared a similar primary specificity in that it cleaved after a P1 Asp residue. Around 24 and 17% of the granzyme B substrates have P1 and P4 Asp residues, respectively. Aside from the P1 site specificity, we noted a modest preference for Glu residues at P3 (from 17 to 52%) and Gly residues at the P1′ position (from 9 to 47%) for both caspases and granzyme B.

Furthermore, upon closer examination, we were able to identify subtle, but important differences in the substrate specificities between different proteases. For example, in addition to the apparent requirement for Asp residues at the P1 and P4 positions, caspase-1 prefers large, hydrophobic amino acids in the P4 position, while for caspase-3, the P4 Asp residue appears to be preferred in most cases for efficient hydrolysis. Substitution of this residue with other amino acids resulted in a >100-fold decrease in the *k*_cat_/*k*_m_ value, indicating the critical importance of having an Asp residue at this position [67]. Comparison of different caspase and granzyme B substrates also revealed distinct patterns of subsite specificities for different enzymes in the P6 to P6′ sites. For caspase-1 substrates, a Ser residue was preferred at P1′, while caspase-3, 7 and 8 substrates tended to have a Gly residue at P1′, with only modest preferences for serine at this position. The differences in substrate specificities between different proteases highlight the necessity to train machine learning models based on their own substrate datasets in order to identify putative family-specific substrates.

### Analysis of structural determinants that characterize the protease substrate specificity

**Figure 4:**
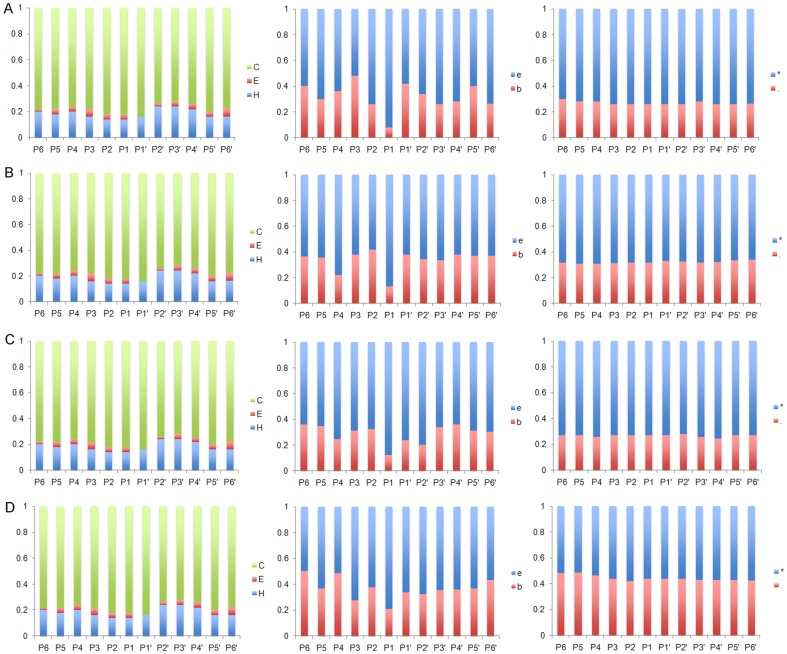
Structural determinants of protease substrate specificity based on occurrences at the P6-P6′ positions for cleavage sites. In each panel, from the left to right, are the assignments at each position for secondary structure (three states: “H”, helix; “E”, strand; “C”, coil), solvent accessibility (two states: “e”, exposed; “b”, buried) and native disorder (two states: “*”, disordered; “.”, ordered), respectively. (A) caspase-1; (B) caspase-3; (C) caspase-7 and (D) caspase-6.

A comprehensive analysis was performed to reveal important structural determinants that characterize the protease substrate specificity, based on the curated substrate datasets. We analyzed the assignments of secondary structure (H, helix; E, strand; C, coil), solvent accessibility (E, exposed; B, buried) and native disorder (“*”, disordered; “.”, ordered) at each position from P6 to P6′. The results for caspase-1, 3, 7 and 6 are shown in Figure 4 A–D, while those for caspase-8, granzyme B (human) and granzyme B (mouse) are shown in Figure S2 E–G.

Previous studies have indicated that certain proteases are more likely to cleave substrates within flexible, solvent-exposed, disordered and secondary structure-depleted regions [27], [68]. Indeed, we note that some proteases frequently cleave substrates within coils or loops, which is consistent with recent proteomics-based profiling studies [7], [14]. Depending on the cleavage site and the protease type, the majority of cleavage sites (72–84%) are observed to be located within predicted coiled regions. However, it is notable that 14–24% of cleavage events take place within α-helices. In contrast a minority of cleavage sites (2–6%) are present in β-strands (Figure 4 and Figure S2). The cleavage of substrates in structural regions such as α-helices and β-strands has been attributed to presence of structural dynamics or conformational switching in these regions upon substrate binding and catalytic hydrolysis by the protease [14], [28], [69]. According to our present understanding of protease-substrate interactions, it would require considerable unfolding for a helical segment to bind into the active sites of a protease in a manner appropriate for catalysis. In addition, the appropriate presentation to the protease of a cleavage site on a solvent accessible surface is a key factor that determines whether a substrate can be accessed and cleaved by the enzyme. A large percentage of cleavage sites (80–92%) are predicted to be solvent accessible (Figure 4), while only a small fraction of cleavage sites (8–20%) are predicted to occur in solvent inaccessible regions.

Natively disordered or unstructured regions have no stable structures without their interaction partners. They are especially abundant in eukaryotic proteomes, where ∼30–60% of eukaryotic proteins are predicted to contain long stretches of natively disordered residues [70], [71]. It is increasingly clear that they are often functionally important and commonly associated with molecular assembly, protein modification, molecular recognition and protein degradation events [72]–[79]. It was shown that cleavage of caspase and granzyme B substrates tends to occur on flexible, disordered regions of substrates [68] and native disorder features have been used to improve the prediction performance of caspase cleavage sites and phosphorylation sites [80]. In this study, we found that the majority of cleavage sites (66–78%) are localized in natively disordered regions. We also performed enrichment analysis of natively disordered residues and solvent exposed residues across different protease substrate types, as shown in Figure S3. Our finding is in agreement with other studies [27], where the amount of predicted disorder in caspase and granzyme B substrates was found to be greater than that in the non-cleaved sequences. All of these results suggest that substantial dynamics in the structure of cleavage sites of protease substrates must occur. Cleavage of substrates within natively disordered regions and outside of structured domains might present potential advantages since less conformational change in the substrate would be required, thus facilitating more efficient hydrolysis of such substrates by proteases.

In summary, there is a clear preference for known cleavage sites (in the P6 to P6′ positions) to be located within looped, solvent accessible and natively disordered regions, which are specified respectively by three different structural features: secondary structure, solvent accessibility and native disorder. The results obtained here highlight the value of using these predicted structural features to further enhance the performance of cleavage site prediction.

### Performance evaluation of PROSPER based on different sequence encoding schemes

**Figure 5:**
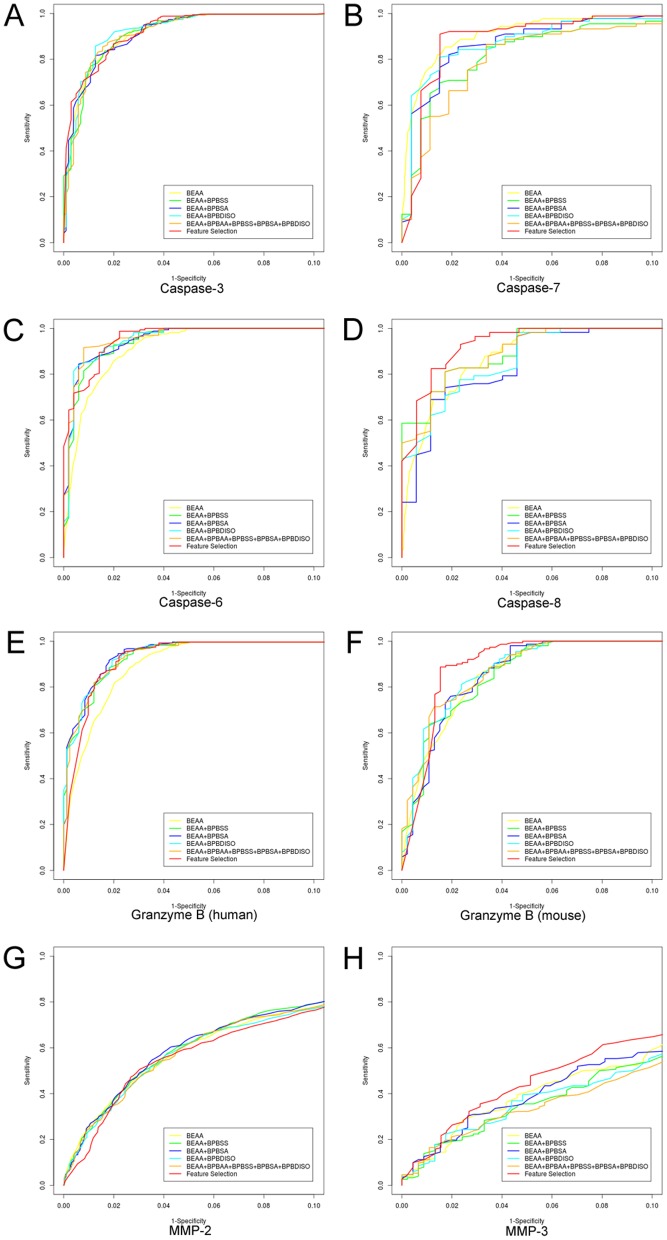
Assessing the performance of PROSPER models for cleavage site prediction of eight proteases, based on gradually increased features to evaluate the relative contribution of each type of feature. For clarity, the ROC curves with high prediction specificities were displayed. Panels A–H correspond to caspase-3, 7, 6, 8, granzyme B (human), granzyme B (mouse), MMP-2 and MMP-3, respectively. Yellow: ROC curves of the trained PROSPER models based on the sequence encoding scheme “BEAA” which includes the binary encoding amino acid sequence profile surrounding the cleavage site; green: ROC curves based on the sequence encoding scheme “BEAA+BPBSS”, which includes the binary encoding amino acid sequence profile plus the bi-profile Bayesian secondary structure profile; blue: ROC curves based on the sequence encoding scheme “BEAA+BPBSA”, which includes the binary encoding amino acid sequence profile plus the bi-profile Bayesian solvent accessibility profile; cyan: ROC curves based on the sequence encoding scheme “BEAA+BPBDISO”, which includes the binary encoding amino acid sequence profile and bi-profile Bayesian native disorder profile; orange: ROC curves based on the sequence encoding scheme “BEAA+BPBAA+BPBSS+BPBSA+BPBDISO”, which includes all of the relevant features; red: ROC curves based on the most informative features as selected by a random forest algorithm.

To evaluate the performance of PROSPER for cleavage site prediction of multiple proteases, we carried out a 5-fold cross-validation test on each type of protease under investigation in this study. We trained PROSPER models based on combinations of sequence and structure profiles with gradually increasing complexity of features, and examined the influences of different feature types on the predictive performance. Table 2 summarizes the performance of PROSPER for cleavage site prediction based on the encoding scheme “BEAA+BPBAA+BPBSS+BPBSA+BPBDISO” using a local window size of P4-P2′. We also assessed the predictive performances of different sequence encoding schemes on cleavage site prediction for different proteases by plotting the ROC (receiver operating characteristic) curves (Figure 5 and Figure S4).

**Table 2:** Performance of PROSPER for predicting cleavage sites of 24 protease families under consideration in this study, measured by Accuracy, Sensitivity, Specificity, F-score and MCC, respectively.

Protease family	Protease	Merops ID	Accuracy (%)	Sensitivity (%)	Specificity (%)	F-score (%)	MCC
**Aspartic protease**	HIV-1 retropepsin	A02.001	85.5	75.0	89.0	72.1	0.678
**Cysteine protease**	Cathepsin K	C01.036	79.6	47.1	90.6	53.7	0.527
	Calpain-1	C02.001	80.2	38.3	94.2	49.2	0.496
	Caspase-1	C14.001	87.5	52.0	99.3	67.5	0.658
	Caspase-3	C14.003	94.6	82.8	98.5	88.5	0.858
	Caspase-7	C14.004	89.6	60.7	99.3	74.5	0.720
	Caspase-6	C14.005	93.7	76.6	99.4	85.9	0.832
	Caspase-8	C14.009	89.7	65.5	97.7	76.0	0.729
**Metalloprotease**	Matrix metallopeptidase-2	M10.003	87.0	77.4	90.2	74.8	0.704
	Matrix metallopeptidase-9	M10.004	81.2	28.9	98.6	43.4	0.463
	Matrix metallopeptidase-3	M10.005	79.9	33.6	95.4	45.5	0.470
	Matrix metallopeptidase-7	M10.008	81.6	31.6	98.2	46.2	0.483
**Serine protease**	Chymotrypsin A (bovine)	S01.001	88.5	79.5	91.5	74.5	0.733
	Granzyme B (human)	S01.010	97.1	96.4	97.3	94.3	0.926
	Elastase-2	S01.131	82.9	37.8	98.0	52.5	0.530
	Cathepsin G	S01.133	81.0	71.6	84.1	65.3	0.613
	Granzyme B (mouse)	S01.136	93.2	80.5	97.4	85.5	0.824
	Thrombin	S01.217	90.2	64.9	98.6	76.7	0.738
	Plasmin	S01.233	87.8	64.6	95.5	72.5	0.691
	Glutamyl peptidase I	S01.269	91.4	84.5	93.7	83.1	0.793
	Furin	S08.071	93.0	72.0	100	83.7	0.811
	Signal peptidase I	S26.001	94.6	82.5	98.6	88.4	0.858
	Thylakoidal processing peptidase	S26.008	89.5	69.8	96.1	76.9	0.738
	Signalase	S26.010	85.8	50.5	97.6	64.0	0.622

Overall, the performances of PROSPER generally increased with the addition of input features to the SVR models. PROSPER models that combined sequence profiles such as “BEAA”, along with other types of structural features usually achieved better results than using the sequence profile alone. In particular, PROSPER achieved the best performance when using the encoding scheme “BEAA+BPBAA+BPBSS+BPBSA+BPBDISO” (for brevity, we call this “ALL” hereafter) [see Table 2 and Tables S1,S2,S3,S4 for performance comparison between different sequence encoding schemes]. Based on these results, it is apparent that a combination of different types of features usually outperformed the individual components alone. This trend can also be seen from the ROC curves (Figure 5 and Figure S4). With the addition of different features, most of the ROC curves based on encoding schemes with more features have higher corresponding AUC values, in contrast to the previous encoding schemes with fewer features (see the ROC curves in Figure 5 and Figure S4). Nevertheless, in the case of matrix metallopeptidase-2 and chymotrypsin A (bovine), performance based on the encoding scheme “ALL” (Table 2) is worse than that based on “BEAA” (Table S1), which possibly means that redundant information exists in the feature sets of these protease substrates.

### Improving the predictive performance by incorporating sequence-derived structural feature

**Figure 6:**
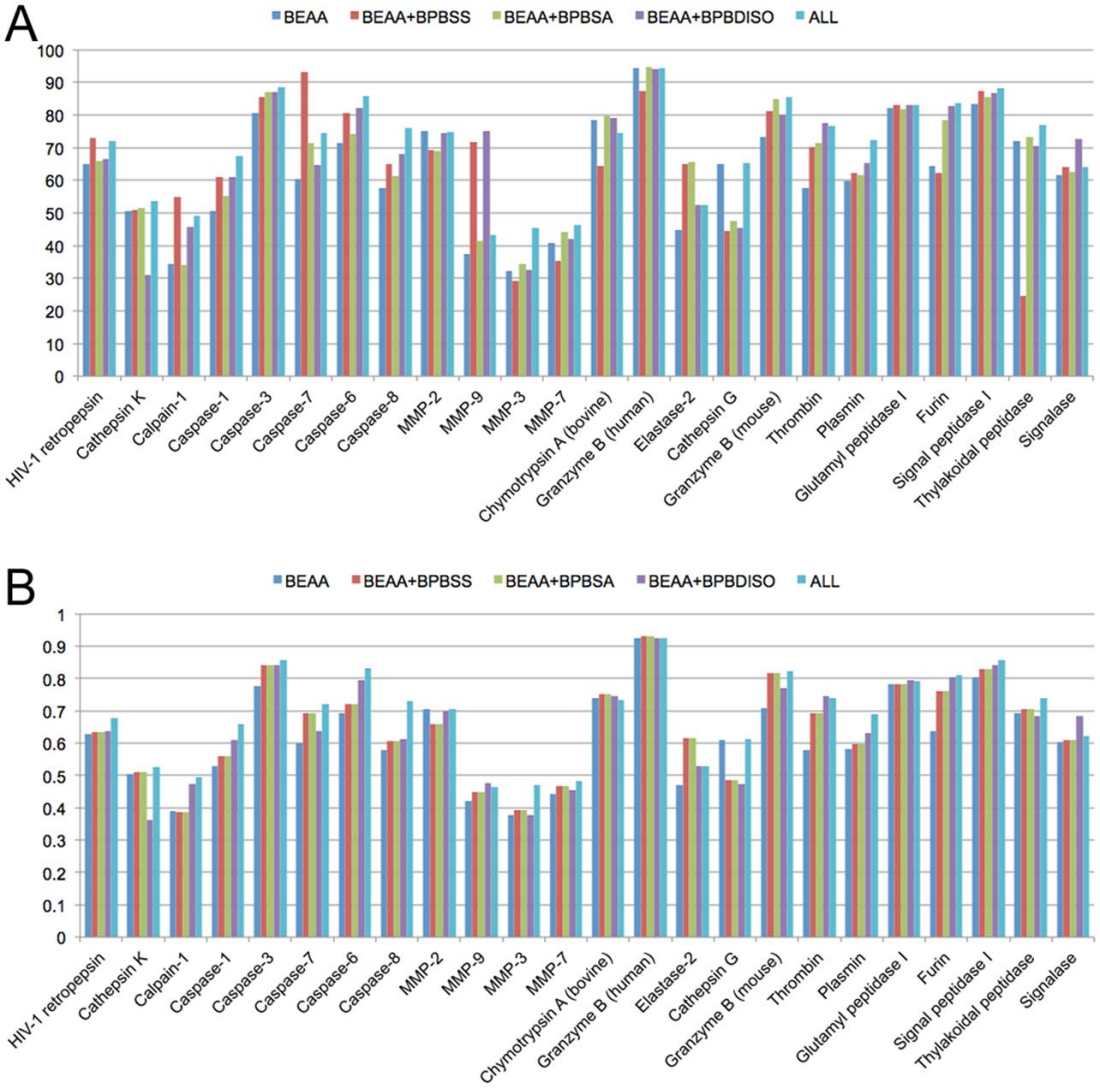
Relative contributions of secondary structure, solvent accessibility and native disorder features to the predictive performance of PROSPER evaluated using F-score (A) and MCC (B), respectively. The predictive performances of four different encoding schemes were compared, including “BEAA”, “BEAA+BPBSA”, “BEAA+BPBDISO” and “ALL”. See the main text for details of different sequence encoding schemes.

We further assessed the relative contributions of secondary structure, solvent accessibility and native disorder features to cleavage site prediction of different proteases by gradually adding each of these feature types into PROSPER models in a step-wise manner and plotting the resulting F-score and MCC measures based on different encoding schemes in Figure 6. The relative contribution of each feature type can be quantified and assessed based on the performance difference between related encoding schemes. As shown in Figure 6, Table S1, S2, S3 and S4, for the majority of the proteases, PROSPER indeed achieved an improved predictive performance after the incorporation of more features, such as measures of secondary structure, solvent accessibility and native disorder. There are six proteases for which PROSPER has achieved satisfactory performance, as judged by having both F-score and MCC values greater than 80%: caspase-3, caspase-6, granzyme B (human), granzyme B (mouse), furin and signal peptidase I. Cleavage sites for the MMP family (MMP-9, MMP-3, and MMP-7) appear to be more difficult to predict, because the F-score and MCC scores for these proteases are smaller than 50%. One important aspect of peptidase specificity that might explain the difficulty in predicting cleavage sites is the importance of exosites. Many proteases have additional binding sites, often on domains other than the protease domain, which effectively restrict the specificity to a very limited number of substrates. The matrix metallopeptidases have hemopexin-like domains that interact with collagens, and the action of thrombin is also limited by an exosite, which may explain why so few cleavage sites were correctly predicted for these proteases. We advise that future efforts be made to better characterize the substrate specificities of these proteases and extract more useful features in order to improve performance in predicting their cleavage sites. For these substrates, exosite binding and interaction (also termed as allosteric regulation) is an absolute requirement in order for the cleavage to occur.

In summary, we conclude that i) incorporation of all relevant features does not necessarily lead to the overall best performance; and ii) it is necessary to be selective about which features to include in the analysis and carefully examine the contribution of each feature type to performance.

### Feature selection by random forest algorithm

**Figure 7:**
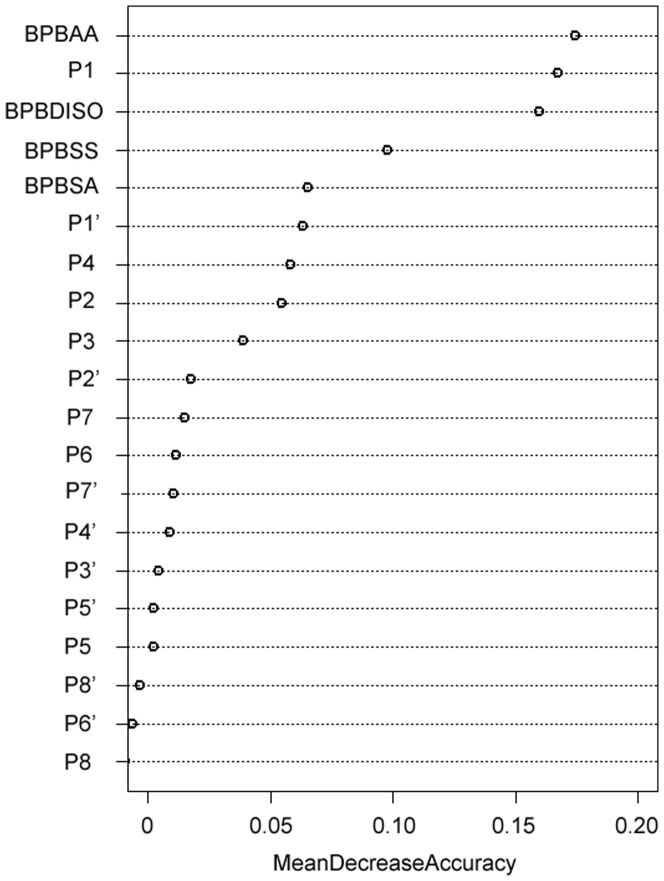
The relative importance of various feature descriptors. Given a local window of P8-P8′, there are twenty different types of feature descriptors in total, including the BEAA profile for each subsite from P8-P8′, and the BPBAA, BPBSS, BPBSA, and BPBDISO profiles. Here, caspase-3 was used as an example to calculate the MeanDecreaseAccuracy value for each feature type based on the feature set.

Since it is likely that sequence-derived features contain redundant information, we carried out feature selection experiments to reduce the initial feature sets by filtering out those features that are regarded as not making a contribution to the predictive performance [81]–[83], as described previously in the Section, *Feature selection*. The random forest algorithm was used to estimate the importance of the twenty different feature types given an entered local window of P8-P8′. Figure 7 shows the relative importance of various feature descriptors for caspase-3 and their contribution to the overall prediction performance. As can be seen from Figure 7, the most important features are BPBAA, P1, BPBDISO, BPBSS, BPBSA, P1′, P4, P2, and P3. The feature selection results for caspase-3 are consistent with the heat map and sequence logo representations of its substrate specificity (shown in Figure 2B and 3B, respectively), because most of the important sequence and structural determinants of its substrate specificity are retained after feature selection. For example, P1, P1′, P4 and P2 are known to play important roles in the substrate selectivity of caspases and they are retained in the final feature sets.

For each protease, features with a Z-score larger than 1.0 were selected as the optimal features to incorporate into the PROSPER models based on sequence encoding scheme “ALL” with feature selection. We then calculated the AUC values from the ROC curves and listed the results in Table S5. After feature selection, approximately 92–99% of the features in the initial feature sets were reduced. However, with the reduced feature sets, we obtained slightly inferior prediction performances and, in some cases, even superior performances with increased AUC values in comparison with the encoding scheme “ALL” without feature selection. This is particularly the case for calpain-1, caspase-3, caspase-7, chymotrypsin A, cathepsin G, granzyme B (mouse), plasmin, thylakoidal processing peptidase and signalase (Table S5). Since different proteases have different substrate specificities, the selected optimal features sets vary from one to another. The full lists of the selected optimal features and all features vectors of the encoding scheme “ALL” are provided in Table S6 and S7, respectively.

### Comparison with other prediction tools

In recent years, several general tools have been developed to predict cleavage sites for various proteases, such as PoPS [32] and SitePrediction [33]. To objectively compare the prediction results, we first tested these three tools on the same training and testing datasets, based in turn on the compiled substrate datasets. Since PoPS and SitePrediction output all of the ranked predicted cleavage sites based on their own selected thresholds, we evaluated their performance by calculating the percentages of correctly predicted cleavage sites in the testing sets. We submitted the substrate datasets to the web servers of PoPS and SitePrediction, analyzed the substrate sequence scanning results and calculated the percentage of correctly predicted cleavage sites by the tools (Table 3).

**Table 3:** Performance comparison of PROSPER with PoPS and SitePrediction for predicting cleavage sites for selected enzymes.

**Caspase-1**						**MMP-2**					
PoPS						PoPS					
Sp (%)	99.9	99.8	99.5	99.0	-a	Sp (%)	99.9	99.8	99.5	99.0	-
Sn (%)	14.0	34.0	48.0	72.0	-	Sn (%)	4.2	5.8	15.4	27.2	-
SitePrediction						SitePrediction					
Sp (%)	99.9	99.8	99.5	99.0	98.0	Sp (%)	99.9	99.8	99.5	99.0	98.0
Sn (%)	**60.0**	**68.0**	72.0	86.0	92.0	Sn (%)	6.7	12.4	22.2	36.4	50.8
PROSPER^5CV^						PROSPER^5CV^					
Sp (%)	99.9	99.8	99.5	98.9	98.0	Sp (%)	99.8	99.7	99.4	99.0	98.1
Sn (%)	12.0	14.0	36.0	56.0	80.0	Sn (%)	8.8	15.3	23.9	31.0	43.8
PROSPER^Select^						PROSPER^Select^					
Sp (%)	99.9	99.8	99.4	99.0	98.0	Sp (%)	99.9	99.7	99.5	99.0	98.1
Sn (%)	22.0	40.0	66.0	80.0	88.0	Sn (%)	**10.3**	17.9	23.0	32.1	39.7
PROSPER^Self^						PROSPER^Self^					
Sp (%)	99.9	99.8	99.5	99.0	98.1	Sp (%)	99.8	99.7	99.4	99.1	98.0
Sn (%)	10.0	36.0	**88.0**	**94.0**	**98.0**	Sn (%)	8.1	**18.0**	**36.0**	**48.2**	**67.5**
**Caspase-3**						**MMP-9**					
PoPS						PoPS					
Sp (%)	99.9	99.8	99.5	99.0	-	Sp (%)	99.9	99.8	99.5	99.2	-
Sn (%)	42.6	50.4	72.3	86.6	-	Sn (%)	2.8	**9.5**	12.8	22.3	-
SitePrediction						SitePrediction					
Sp (%)	99.9	99.8	99.5	99.0	98.0	Sp (%)	99.9	99.8	99.5	99.0	98.0
Sn (%)	**52.5**	62.7	81.6	91.2	97.4	Sn (%)	2.4	7.6	14.7	21.3	41.7
PROSPER^5CV^						PROSPER^5CV^					
Sp (%)	99.9	99.7	99.5	99.0	98.0	Sp (%)	99.9	99.8	99.5	99.0	98.0
Sn (%)	28.6	56.3	68.2	81.9	93.6	Sn (%)	2.4	6.2	12.3	20.4	29.9
PROSPER^Select^						PROSPER^Select^					
Sp (%)	99.8	99.7	99.5	99.0	98.0	Sp (%)	99.9	99.8	99.5	99.0	98.0
Sn (%)	51.3	66.2	76.7	86.0	93.6	Sn (%)	3.3	6.2	10.4	17.1	28.4
PROSPER^Self^						PROSPER^Self^					
Sp (%)	99.9	99.8	99.5	99.0	98.0	Sp (%)	99.9	99.8	99.5	98.9	98.0
Sn (%)	30.0	**76.1**	**92.4**	**97.4**	**99.4**	Sn (%)	**3.8**	**9.5**	**28.4**	**48.8**	**61.6**
**Caspase-7**						**GrB (human)**					
PoPS						PoPS					
Sp (%)	99.9	99.8	99.5	99.0	-	Sp (%)	99.9	99.8	99.5	99.0	-
Sn (%)	56.2	65.2	79.8	93.3	-	Sn (%)	17.4	23.6	34.1	43.8	-
SitePrediction						SitePrediction					
Sp (%)	99.9	99.8	99.5	99.0	98.0	Sp (%)	-	-	-	-	-
Sn (%)	57.3	66.3	76.4	89.9	96.6	Sn (%)	-	-	-	-	-
PROSPER^5CV^						PROSPER^5CV^					
Sp (%)	99.9	99.8	99.5	99.0	98.0	Sp (%)	99.9	99.8	99.6	99.0	98.0
Sn (%)	33.7	49.4	75.3	85.4	87.6	Sn (%)	19.9	37.0	53.3	67.8	83.0
PROSPER^Select^						PROSPER^Select^					
Sp (%)	99.9	99.8	99.5	99.0	98.0	Sp (%)	99.9	99.8	99.5	99.0	98.0
Sn (%)	40.4	58.4	84.3	87.6	89.9	Sn (%)	12.7	24.3	35.9	55.1	71.0
PROSPER^Self^						PROSPER^Self^					
Sp (%)	100	99.8	99.5	99.1	98.1	Sp (%)	99.9	99.8	99.5	99.0	98.0
Sn (%)	**61.8**	**97.8**	**100**	**100**	**100**	Sn (%)	**23.9**	**47.5**	**83.7**	**93.8**	**98.6**
**Caspase-6**						**GrB (mouse)**					
PoPS						PoPS					
Sp (%)	99.9	99.8	99.5	99.0	-	Sp (%)	99.9	99.8	99.5	99.0	-
Sn (%)	**20.4**	23.4	44.9	68.3	-	Sn (%)	14.9	20.5	33.6	50.8	-
SitePrediction						SitePrediction					
Sp (%)	99.9	99.8	99.5	99.0	98.0	Sp (%)	-	-	-	-	-
Sn (%)	**20.4**	37.1	**80.2**	**92.8**	95.2	Sn (%)	-	-	-	-	-
PROSPER^5CV^						PROSPER^5CV^					
Sp (%)	99.9	99.8	99.4	99.0	98.0	Sp (%)	99.9	99.8	99.5	98.9	97.9
Sn (%)	15.0	25.8	53.3	66.5	83.8	Sn (%)	21.4	34.4	45.5	55.8	73.4
PROSPER^Select^						PROSPER^Select^					
Sp (%)	99.9	99.8	99.5	98.9	98.1	Sp (%)	99.9	99.8	99.5	99.1	98.1
Sn (%)	16.8	29.9	55.7	77.8	92.8	Sn (%)	18.2	29.2	46.8	63.6	79.2
PROSPER^Self^						PROSPER^Self^					
Sp (%)	99.9	99.8	99.5	99.0	98.1	Sp (%)	99.9	99.8	99.5	99.0	98.0
Sn (%)	15.6	**50.9**	68.3	84.4	**98.8**	Sn (%)	**27.9**	**54.6**	**77.9**	**92.9**	**98.0**
**Caspase-8**											
PoPS											
Sp (%)	99.9	99.8	99.5	99.0	-						
Sn (%)	**48.3**	60.4	77.6	91.4	-						
SitePrediction											
Sp (%)	99.9	99.8	99.5	99.0	-						
Sn (%)	**48.3**	**69.0**	89.7	96.6	-						
PROSPER^5CV^											
Sp (%)	99.9	99.8	99.5	99.0	98.0						
Sn (%)	22.4	36.2	46.6	69.0	77.6						
PROSPER^Select^											
Sp (%)	99.9	99.8	99.6	99.0	98.0						
Sn (%)	25.9	51.2	62.1	84.5	91.4						
PROSPER^Self^											
Sp (%)	99.9	99.8	99.5	99.0	98.0						
Sn (%)	13.8	41.4	**91.4**	**98.3**	**98.3**						

a “-” denotes that the prediction result at this specificity level is not available by this tool.

Specificity (Sp) levels were set as close as possible to one another among the different tools. For PROSPER, 5-fold cross-validation ± feature selection and self-consistency tests were performed to compare to PoPS and SitePrediction. The 3 different types of PROSPER models built are named PROSPER^5CV^, PROSPER^select^ and PROSPER^Self^, respectively. Sensitivity (Sn) values at different Sp levels were compared. The best prediction performance at each Specificity level is highlighted in bold.

PoPS is a comprehensive bioinformatics tool for modelling and predicting substrate cleavage sites for various proteases [32] (http://pops.csse.monash.edu.au/). It allows users to build computational models of protease substrate specificity that can be used to predict and rank potential cleavage sites for the protease of interest. SitePrediction is another general tool for predicting substrate cleavage sites of proteases [33] (http://www.dmbr.ugent.be/prx/bioit2-public/SitePrediction/). It combines the amino acid frequency score with an amino acid substitution matrix score that indicates the similarity of the potential cleavage sites to the known cleavage sites. The final score is calculated as the product of these two scores. In contrast to PROSPER, which was developed based on machine learning techniques, both PoPS and SitePrediction are regarded as empirical scoring-based prediction tools, which makes it particularly interesting to compare the performance of different types of methods.

Since all of the compared tools have pre-defined thresholds to select predicted cleavage sites, we adjusted the different Specificity levels as close as possible to 99.9, 99.8, 99.5, 99.0 and 98.0% and compared the corresponding sensitivities. This comparison strategy has been suggested in previous studies [80]. In order to comprehensively evaluate the performance of PROSPER with other prediction tools, we trained PROSPER models without and with feature selection based on 5-fold cross-validation and self-consistency tests. The corresponding PROSPER models are termed PROSPER^5CV^, PROSPER^select^ and PROSPER^Self^, respectively, in Table 3 below. It can be seen from Table 3 that PROSPER^Self^ achieved higher sensitivity in most cases when compared with PoPS and SitePrediction. Another finding is that PROSPER^select^ based on feature selection performed much better than PROSPER^5CV^ without feature selection, indicating the importance of efficient feature selection to improved prediction performance. In most cases, SitePrediction achieved greater sensitivity at the given specificity level compared to PoPS, especially for caspases. This can be explained by the fact that SitePrediction combines the use of a frequency score that indicates whether the amino acids of potential cleavage sites are likely to occur at the position and an amino acid substitution matrix that indicates the similarity of the potential cleavage sites [33], while PoPS relies on a position-specific scoring matrix (PSSM) based on amino acid frequency to build predictive models [32]. Altogether, the prediction performance of PROSPER is at least comparable to the other two tools.

**Figure 8:**
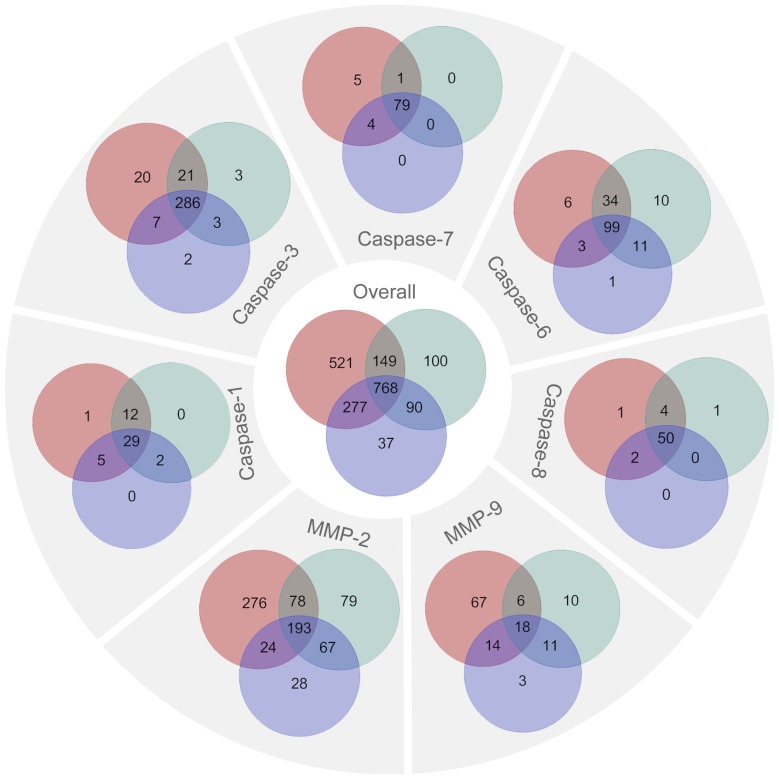
Venn diagrams showing the prediction consistency between PROSPER, PoPS and SitePrediction. The different colored circles denote different prediction tools: PROSPER, red; PoPS, blue; SitePrediction, green. The sum of the numbers in each color represents the number of known cleavage sites that were correctly predicted by the tool. The number in each overlapping region represents the number of known cleavage sites that were correctly predicted by two or all tools. For example, for MMP-2, 276 sites were correctly predicted by PROSPER and by no other tool, 78 were correctly predicted by PROSPER and SitePrediction, 24 by PROSPER and PoPS and 193 by all three tools. The number in the inner circle “Overall” represents the total number of correctly predicted known cleavage sites by the corresponding tool.

The prediction consistency among the different tools is shown in Figure 8. Venn diagrams show the distribution of correctly predicted cleavage sites: 768 known cleavage sites were correctly predicted by all three tools; 277 known cleavage sites were correctly predicted by both PROSPER and PoPS; 149 known cleavage sites were correctly predicted by both PROSPER and SitePrediction, while 90 were correctly identified by PoPS and SitePrediction. The family-specific distributions showed that the numbers of correctly predicted known cleavage sites by PROSPER, in most cases, were higher than those predicted by PoPS and SitePrediction. Nevertheless, there are also significant number of known cleavage sites that were correctly predicted by PoPS and SitePrediction, yet were not identified by PROSPER. This suggests that a meta or consensus approach could potentially be developed to make a better prediction by integrating the prediction results of all three tools.

Moreover, we further tested the predictive powers of these three tools to recognize novel protease substrates by performing an independent test based on cleavage sites of protease substrates that were recently experimentally verified, as well as using the recent update of MEROPS. Due to limitations in data availability, we could only perform a comparison for four proteases: caspase-3, MMP-2, granzyme B (human) and granzyme B (mouse). The performance comparison shown in Table 4 indicates that PROSPER yielded higher accuracies than PoPS and SitePrediction, except in the case of caspase-3, for which PoPS achieved a 9% higher accuracy.

**Table 4:** Performance comparison of PROSPER with PoPS and SitePrediction based on independent test datasets extracted from recent proteomics profiling studies and a recent update of the MEROPS database.

Protease	Merops ID	Percentage of correctly predicted known cleavage sites (%)		
		PoPS	SitePrediction	PROSPER
**Caspase-3** a	C14.003	92.7	45.6	83.6
**Matrix metallopeptidase-2** b	M10.003	17.6	3.9	29.4
**Granzyme B (human)** a	S01.010	47.8	-c	76.9
**Granzyme B (mouse)** a	S01.136	65.1	-	77.4

a Substrate datasets extracted from a recent update of the MEROPS database [34], [35];

b Substrate dataset extracted from experimental data derived from N-terminal positional proteomics [20];

c “-” denotes that the prediction result for this protease family is not available for this tool.

Note that these data are only available for a few proteases, including caspase-3, MMP-2, granzyme B (human) and granzyme B (mouse). The accuracy or sensitivity was calculated as the percentage of the known cleavage sites that were correctly predicted.

### Proteome-wide substrate cleavage site prediction

We applied PROSPER with a high stringency at 100% Specificity level to scan the human and mouse proteomes extracted from the IPI database [84], which have 87,040 and 56,687 proteins, respectively (note that the IPI database includes splice variants, thus the number of human proteins is much greater than the number of human genes mentioned previously). Since caspase-1, 3, 7, 6, 8, granzyme B (human) and granzyme B (mouse) represent proteases with well-known substrate specificities and the performances of PROSPER for their cleavage sites prediction are more accurate compared to other proteases, we applied their respective PROSPER models to scan the whole human and mouse proteomes to identify putative cleavage sites, resulting in many predictions with high-confidence scores.

The statistics of predicted cleavage sites are shown in Table 5. The distribution of Gene Ontology assignments [85] for the predicted substrates can be seen in Figure S5 and all the predictions are available at http://lightning.med.monash.edu.au/PROSPER/. Taking caspase-3 as an example, membrane, nucleus and cytoplasm were the three largest categories containing predicted caspase-3 substrates and account for 38, 17 and 10% of the annotations, respectively. Intracellular, mitochondrion, golgi apparatus and cytosol represent 9, 3, 3 and 3% of the annotations, with the final 14% of annotations split between the remaining biological process categories. To further investigate the function of the potential substrates of caspase-3 (and other proteases), we used ToppFun, which is a gene list enrichment analysis and candidate gene prioritization tool [86]. The results indicate that the majority of the predicted caspase-3 substrates (using the human genome as control) have molecular functions such as enzyme binding, nucleoside-triphosphatase, GTPase regulator, pyrophosphatase, hydrolase, transferase, kinase and phosphotransferase activity (Table 6). Our analysis also revealed that most of the predicted substrates are involved in biological processes such as cell projection organization, cell adhesion, nucleotide catabolic processes, neurogenesis, etc. (Table 6). In addition, we found that the majority of the predicted substrates of caspase-3 have cellular components in compartments such as cell projections, nucleoplasm, cytoskeleton, cell junction, synapse, etc. The significantly enriched GO terms of the predicted substrates of other proteases that are available to be analyzed by gene list enrichment analysis can be found in Table S8.

**Table 5:** Proteome-wide substrate cleavage site predictions at the 100% Specificity level by PROSPER.

Protease	Merops ID	Number of predicted cleavage sites (Human proteome)	Number of predicted cleavage sites (Mouse proteome)
**Caspase-1**	C14.001	592,172	44,0217
**Caspase-3**	C14.003	3,339	2,425
**Caspase-7**	C14.004	22,322	16,590
**Caspase-6**	C14.005	58,021	43,778
**Caspase-8**	C14.009	117,891	87,109
**Granzyme B (human)**	S01.010	127,045	93,043
**Granzyme B (mouse)**	S01.136	57,149	40,935

This specificity level was used to generate high-confidence prediction results.

**Table 6:** The significantly enriched Gene Ontology (GO) terms of the predicted caspase-3 substrates.

Gene Ontology category	Rank	ID	Name	P-value	Term in predicted substrates	Term in human Genome
Molecular Function	1	GO:0019899	enzyme binding	9.166E-23	677	834
	2	GO:0017111	nucleoside-triphosphatase activity	3.865E-22	627	768
	3	GO:0030695	GTPase regulator activity	1.187E-20	397	464
	4	GO:0016462	pyrophosphatase activity	2.433E-20	645	799
	5	GO:0016818	hydrolase activity, acting on acid anhydrides, in phosphorus-containing anhydrides	6.651E-20	646	802
	6	GO:0016817	hydrolase activity, acting on acid anhydrides	9.754E-20	647	804
	7	GO:0016772	transferase activity, transferring phosphorus-containing groups	4.995E-19	775	984
	8	GO:0016301	kinase activity	6.297E-19	681	854
	9	GO:0016773	phosphotransferase activity, alcohol group as acceptor	2.039E-18	588	728
	10	GO:0060589	nucleoside-triphosphatase regulator activity	2.880E-18	402	477
Biological Process	1	GO:0030030	cell projection organization	8.327E-25	640	776
	2	GO:0007155	cell adhesion	2.443E-22	713	884
	3	GO:0022610	biological adhesion	2.443E-22	713	884
	4	GO:0006195	purine nucleotide catabolic process	2.949E-19	449	536
	5	GO:0072523	purine-containing compound catabolic process	6.385E-19	452	541
	6	GO:0009203	ribonucleoside triphosphate catabolic process	1.148E-18	411	487
	7	GO:0009154	purine ribonucleotide catabolic process	1.156E-18	414	491
	8	GO:0009207	purine ribonucleoside triphosphate catabolic process	1.509E-18	410	486
	9	GO:0022008	neurogenesis	2.076E-18	796	1015
	10	GO:0009261	ribonucleotide catabolic process	3.434E-18	416	495
Cellular Component	1	GO:0042995	cell projection	1.922E-21	835	1069
	2	GO:0005654	nucleoplasm	5.390E-19	1018	1341
	3	GO:0015630	microtubule cytoskeleton	9.487E-18	552	689
	4	GO:0044451	nucleoplasm part	3.247E-14	608	782
	5	GO:0030054	cell junction	7.553E-13	494	628
	6	GO:0045202	synapse	2.918E-12	380	472
	7	GO:0005626	insoluble fraction	5.411E-12	830	1111
	8	GO:0044430	cytoskeletal part	6.123E-12	836	1120
	9	GO:0005624	membrane fraction	3.573E-11	796	1067
	10	GO:0005730	nucleolus	2.223E-10	838	1133

The significantly enriched GO terms of the predicted caspase-3 substrates according to three major categories (Molecular Function, Biological Process and Cellular Component) were obtained using the gene list enrichment analysis tool, ToppFun [86]. The *P*-value of each GO term in the predicted substrates was calculated by randomly sampling the whole genome.

For a particular protease of interest, users can further filter out the false positives and only retain ‘meaningful’ predictions. By ‘meaningful’ predictions, we mean that the protease and the predicted substrates should in principle share the same subcellular localizations so that they can co-localize *in vivo* in order for the substrate cleavage to occur, which can be accomplished according to the accompanied GO annotations. In this sense, these predictions provide a valuable resource for further experimental validation of novel protease substrates and the proposition of useful hypotheses.

### The implementation of PROSPER webserver

**Figure 9:**
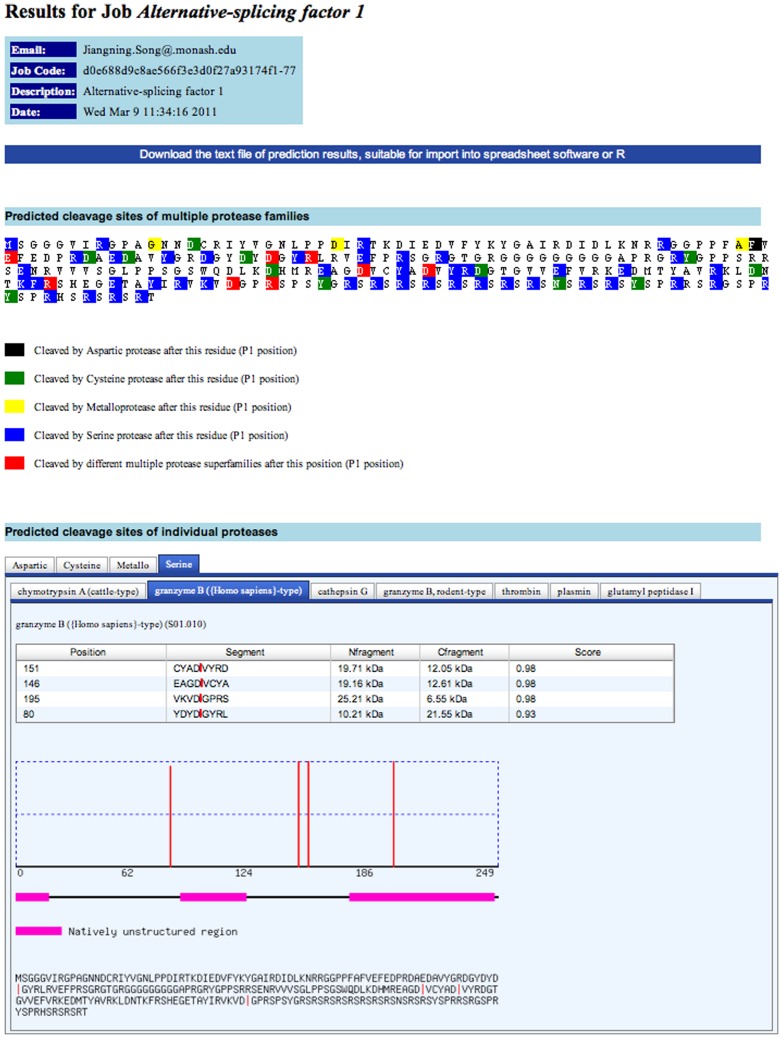
A sample output from the PROSPER server for the substrate, serine/arginine-rich splicing factor 1 (SRSF1) (Uniprot ID: Q07955).

The online webserver of PROSPER has been implemented as a result of this work and has been made publicly available at http://lightning.med.monash.edu.au/PROSPER/ for academic users. The server accepts a single amino acid sequence in the FASTA format as an input. After job submission, the server will first run a few programs to extract the sequence and structural features and then generate the SVM input file, which will be further submitted to the PROSPER models to make predictions. We also made available a job queue processing system, so that each of the multiple tasks submitted simultaneously to the server can be processed one by one in a timely manner. Once the task is completed, users will receive a notification Email with a link to the result webpage. It contains the ranking of predicted cleavage sites according to the cleavage probability scores, the P4-P4′ sequence, the estimated sizes of the cleavage products, and the native disorder plot (Figure 9). The PROSPER server is currently configured on an Intel i7 920 processor with eight cores, running Unbuntu 9.10, with a 12GB memory and 4TB hard-disk. The server scripts are written in Perl. Although the calculation time is dependent on the length of the submitted sequence, a typical task for a query sequence with ∼500 residues will take approximately 8–12 minutes.

With the recent advancement of N-terminal labeling and positional proteomics approaches, the substrate data for a number of proteases is accumulating rapidly. In addition to the online web server, we are currently in the process of implementing a stand-alone version of PROSPER based on Java programming language, which will allow users to build their own customized prediction models based on substrate sets specified by users.

### Case studies

We illustrate the predictive power of PROSPER by performing a case study where proteolytic cleavage of the protein, huntingtin (Htt), by caspase-3 and caspase-6 was examined. The Htt protein plays a critical role in nerve cell function and regularly interacts with proteins found only in the brain. Mutant Htt is highly variable due to the polyglutamine-expansion in its N-terminus [87]–[89]. Proteolysis of Htt at specific residue positions has been recently found to be critical to the pathogenesis of the disease [88], [90]. Experimental studies have indicated that Htt contains four experimentally verified cleavage sites for caspase-3: DSVD|LASC (Position: 513), DEED|ILSH (Position: 530), DLND|GTQA (Position: 552) and IVLD|GTDN (Position: 586), and one cleavage site for caspase-6: IVLD|GTDN 586.

**Figure 10:**
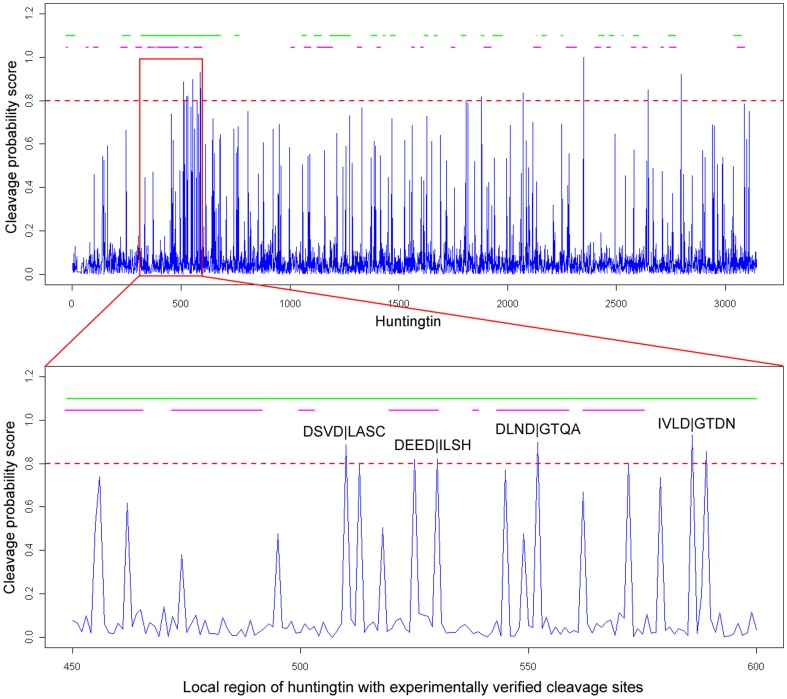
Full-length sequence scanning of huntingtin protein by PROSPER for caspase-3 cleavage sites, with the zoomed-in view of the region flanking experimentally verified cleavage sites of huntingtin. The horizontal axis represents the amino acid position; the vertical axis represents the cleavage probability score generated by PROSPER. The predicted solvent exposed and natively disordered regions on the top of each figure are highlighted by green and red, respectively. A higher threshold value of 0.8 for making the positive cleavage site prediction is denoted by a dashed red line. P4-P4′ sites of the experimentally verified cleavage sites are labeled.

We performed substrate sequence scanning using PROSPER, PoPS and SitePrediction to predict the potential cleavage sites for both caspase-3 (Figure 10) and caspase-6 (Table S9) for Htt. All four experimentally verified cleavage sites for caspase-3 in Htt were correctly predicted and were within the top 20 ranking hits for all three tools. Another experimentally verified cleavage site for caspase-6 was also correctly predicted (among the top 20 hits). In the case of PROSPER, the highest ranking result for one of the known caspase-3 cleavage sites was for DEED|ILSH, with a ranking of fifth place, whereas the other three known cleavage sites ranked 18^th^ to 20^th^. PoPS and SitePrediction also included three common cleavage sites in their lists: DSVD|LASC, DEED|ILSH and DLND|GTQA. Altogether, these results suggest that *in silico* sequence scanning of substrates is helpful for identifying putative cleavage sites.

## Conclusions

Predicting putative protease substrates is a critical step towards better understanding of protease systems biology and enhancing our capability for the design of novel inhibitors as therapeutics to control and regulate protease functions. The recent data accumulation regarding substrate cleavage sites of proteases has increased the demand for efficient bioinformatic approaches that are capable of accurately predicting substrate cleavage sites of proteases. Here we have presented PROSPER, a novel bioinformatics tool which has formulated cleavage site prediction as a binary classification problem and solved it using a machine learning algorithm. The tool has taken advantage of the excellent generalization abilities of machine learning techniques to capture the key characteristics underlying complex protease-substrate interactivity by using kernel functions to build predictive models. The tool, especially when used with efficient feature selection, has been shown to be robust and high performing here using rigorous, independent evaluation protocols. In comparison to existing tools, such as PoPS and SitePrediction, PROSPER achieved at least comparable sensitivity at the varying specificity levels. Further, with the improved performance of PROSPER, we applied it to perform proteome-wide predictions of cleavage sites in the human and mouse proteomes for caspase-1, 3, 7, 6, 8, granzyme B (human) and granzyme B (mouse), resulting in many predictions with high-confidence scores. Due to the limited availability of substrate data which meets the rigorous demands that we have set for inclusion, at present PROSPER is only able to predict the cleavage sites of twenty-four different proteases (Table 1). With the increasing availability of high-quality substrate specificity data [4], [5], [34], [35], it will now be possible to improve the quality of predictive models and regularly update the PROSPER models and make available prediction models for other proteases.

There are a number of ways to improve predictive performance in the future. Firstly, more informative and complementary features surrounding the potential cleavage sites can be incorporated. For example, sequence features that are descriptive of sequence-order context might be helpful to identify cleavage sites of proteases that cleave substrates in a cooperative manner [91], [92]. Secondly, high resolution structures showing the active site of the protease complexed with the corresponding P4-P4′ residues of substrates could be used to predict the preference each subsite has for a particular amino acid residue [8]. Such atomic-level structural modelling of the protease-substrate interaction would most likely help to reduce the significant number of false positives. Thirdly, improving the representation of ‘true negatives’, i.e. sites that really cannot be cleaved under any given physiological conditions [28], could provide better represented positive and negative datasets, allowing optimal sequence and structural feature selection that can be performed to further improve the prediction accuracy of the predictors. Finally, performance improvement can possibly be achieved by utilizing ensemble learning approaches or meta approaches that combine multiple independent basic classifiers to perform a final consensus prediction [93]–[95]; this might be useful to further enhance prediction accuracy. On the other hand, it is important to note that failure of active site-based methods can be an important indicator that other factors, such as exosites, are important for a particular protease and this can provide an important indication to researchers that they need to consider regions outside of the active site in further research on the enzyme.

Characterizing the protease substrate specificity and understanding the underlying mechanisms for cleaving multiple *in vivo* substrates is a common practice in protease systems biology today. *In silico* prediction of substrate cleavage sites could provide valuable insights with regard to the identification of novel protease substrates and hypothesis-driven experimentation within the context of proteolytic pathways. To our knowledge, PROSPER is the first comprehensive server capable of predicting cleavage sites of multiple proteases within a single substrate sequence using machine learning techniques. The PROSPER server provides a user friendly interface and only requires a single amino acid sequence of the substrate as an input and an Email address of the user to send the prediction result webpage. In addition, we also make available a stand-alone version and the source code of PROSPER for download such that bioinformaticians and computational biologists can run predictions of multiple sequences locally. Finally, we anticipate PROSPER to be a powerful bioinformatics tool to mine the repertoire of protease substrates and facilitate the discovery of novel substrates.

## Supporting Information

Figure S1 Sequence logo representations of the occurrences of amino acid residues in the substrate cleavage site P8-P8′ positions. To better reflect the occurrence rate of each amino acid type, the sequence logo ordinates have been scaled in bits (Schneider and Stephens, 1990). Panels A–P correspond to: A, HIV-1 retropepsin; B, cathepsin K; C, calpain-1; D, MMP-9; E, MMP-3; F, MMP-7; G, chymotrypsin A (bovine); H, elastase-2; I, cathepsin G; J, thrombin; K, plasmin; L, glutamyl peptidase I; M, furin; N, signal peptidase I; O, thylakoidal processing peptidase; and P, signalase, which are presented according to the alphabetical order of their MEROPS ID in Table 1.
(TIF)

Figure S2 Analysis of structural determinants of protease substrate specificity based on the occurrences in P6-P6′ positions for cleavage sites. In each panel, from the left, middle to right, are the distributions of secondary structure (three states: “H”, helix; “E”, strand; “C”, coil), solvent accessibility (two states: “e”, exposed; “b”, buried) and native disorder (two states: “*”, disordered; “.”, ordered), respectively. (E) caspase-8; (F) granzyme B (human); (G) granzyme B (mouse).
(TIF)

Figure S3 Enrichment analysis of natively disordered residues and solvent exposed residues across different protease substrate types. Left: protease substrate categories that are enriched in natively disordered residues; Right: protease substrate categories that are enriched in solvent exposed residues. Higher percentage on the x-axis indicates greater enrichment of either native disorder or solvent accessibility.
(TIF)

Figure S4 Assessing the performance of PROSPER models for cleavage site prediction of the 16 proteases, based on gradually increased features to evaluate the relative contribution of each type of feature. Panels A–P correspond to: A, HIV-1 retropepsin; B, cathepsin K; C, calpain-1; D, MMP-9; E, MMP-3; F, MMP-7; G, chymotrypsin A (bovine); H, elastase-2; I, cathepsin G; J, thrombin; K, plasmin; L, glutamyl peptidase I; M, furin; N, signal peptidase I; O, thylakoidal processing peptidase; and P, signalase, which are presented according to the alphabetical order of their MEROPS ID in Table 1. For clarity, the ROC curves with high prediction specificities (90–100%) were displayed.
(TIF)

Figure S5 Distribution of the Gene Ontology annotations of the predicted protease substrates. A) caspase-1; B) caspase-3; C) caspase-7, D) caspase-6, E) caspase-8, F) granzyme B (human), G) granzyme B (mouse), and H) the background distribution based on the whole human proteome.
(TIF)

Table S1 Predictive performance based on singe sequence inputs only (sequence encoding scheme “BEAA”), with the local window size of P4-P2′. The results were obtained by 5-fold cross-validation tests.
(DOC)

Table S2 Predictive performance based on singe sequence inputs only (sequence encoding scheme “BEAA+BPBSS”), with the local window size of P4-P2′. The results were obtained by 5-fold cross-validation tests.
(DOC)

Table S3 Predictive performance based on singe sequence inputs only (sequence encoding scheme “BEAA+BPBSA”), with the local window size of P4-P2′. The results were obtained by 5-fold cross-validation tests.
(DOC)

Table S4 Predictive performance based on singe sequence inputs only (sequence encoding “BEAA+BPBDISO”), with the local window size of P4-P2′. The results were obtained by 5-fold cross-validation tests.
(DOC)

Table S5 The AUC (area under ROC curve) values for PROSPER models based on different sequence encoding schemes: “BEAA”, “BEAA+BPBAA+BPBSS+BPBSA+BPBDISO” (termed as “ALL” here) without feature selection, and “BEAA+BPBAA+BPBSS+BPBSA+BPBDISO with feature selection. An extended local window of P8-P8′ was used to build the PROSPER models. See the main text for details of different sequence encoding schemes.
(DOC)

Table S6 List of the more informative features selected using random forest algorithm. Features with a Z score greater than 1.0 are selected and considered to be more informative. An extended local window size of P8-P8′ was used to perform feature selection in order to extract more relevant features.
(DOC)

Table S7 The numbering and categorization of all feature vectors in the encoding scheme “ALL”. An extended local window size of P8-P8′ using the sequence encoding scheme “ALL” was used to perform feature selection in order to extract more relevant features.
(DOC)

Table S8 The significantly enriched Gene Ontology (GO) terms of the predicted substrates of caspase-1, 7, 6, 8, granzyme B (human) and granzyme B (mouse) that were available to be analyzed by the gene list enrichment analysis tool ToppFun. The significantly enriched GO terms of the predicted substrates are listed according to three major categories: Molecular Function, Biological Process and Cellular Component. The *P*-value of each GO term in the predicted substrates was calculated by randomly sampling the whole genome.
(DOC)

Table S9 Summary of the caspase-3 cleavage site prediction by PROSPER for huntingtin, compared with the prediction results by PoPS and SitePrediction, respectively. The top 20 ranking results of these three tools are listed, where experimentally verified cleavage sites are colored by black and bold. The cleavage score of PROSPER was generated by the regression models of PROSPER. Cleavage score of PoPS is calculated as a summation of individual scores of the P4, P3, P2, P1 and P1′ positions. The final cleavage score of SitePrediction is calculated as the product of both the frequency and similarity scores. The higher the cleavage score, the more likely a cleavage site is predicted to be cleaved. Here, “**|**” indicates the substrate cleavage site after the P1 position.
(DOC)
