# Supplementary figures and images for: PROSPER: An Integrated Feature-Based Tool for Predicting Protease Substrate Cleavage Sites
Source: PLoS One. 2012 Nov 29;7(11):e50300. doi: 10.1371/journal.pone.0050300 (PMC3510211; doi:10.1371/journal.pone.0050300)

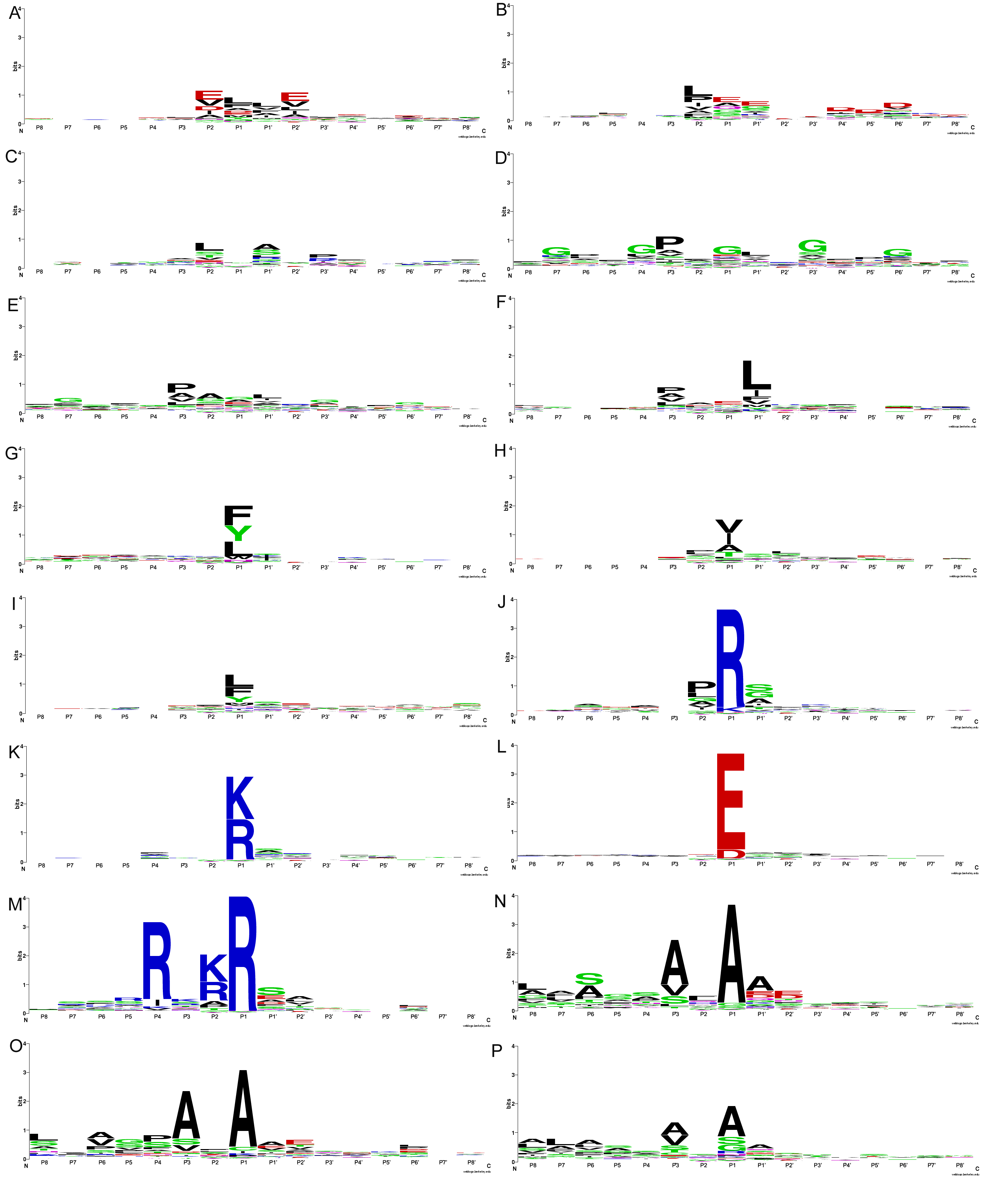

Supplement: Figure S1 — Sequence logo representations of the occurrences of amino acid residues in the substrate cleavage site P8-P8′ positions. To better reflect the occurrence rate of each amino acid type, the sequence logo ordinates have been scaled in bits (Schneider and Stephens, 1990). Panels A–P correspond to: A, HIV-1 retropepsin; B, cathepsin K; C, calpain-1; D, MMP-9; E, MMP-3; F, MMP-7; G, chymotrypsin A (bovine); H, elastase-2; I, cathepsin G; J, thrombin; K, plasmin; L, glutamyl peptidase I; M, furin; N, signal peptidase I; O, thylakoidal processing peptidase; and P, signalase, which are presented according to the alphabetical order of their MEROPS ID in Table 1. (TIF) [file pone.0050300.s001.tif]

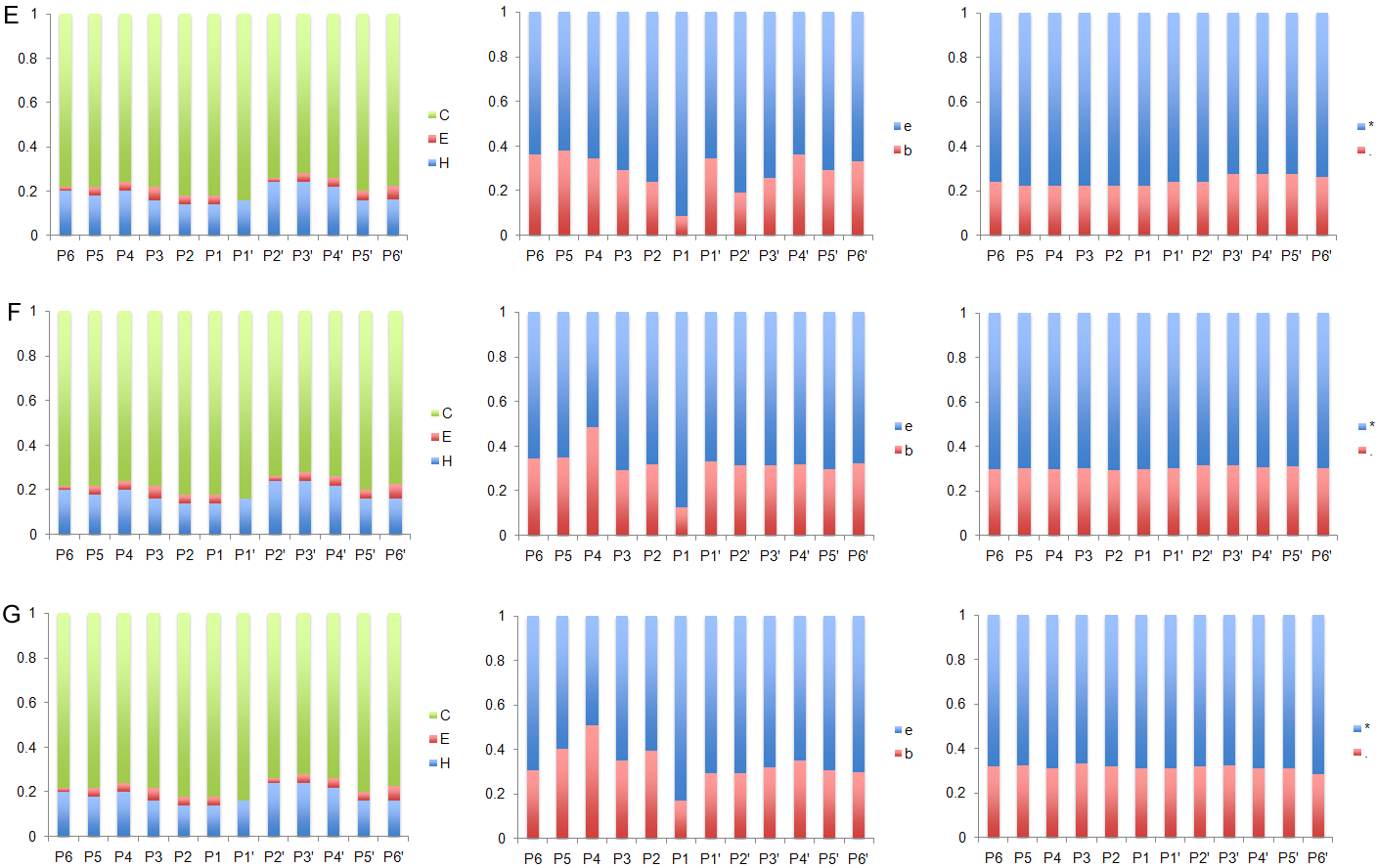

Supplement: Figure S2 — Analysis of structural determinants of protease substrate specificity based on the occurrences in P6-P6′ positions for cleavage sites. In each panel, from the left, middle to right, are the distributions of secondary structure (three states: “H”, helix; “E”, strand; “C”, coil), solvent accessibility (two states: “e”, exposed; “b”, buried) and native disorder (two states: “*”, disordered; “.”, ordered), respectively. (E) caspase-8; (F) granzyme B (human); (G) granzyme B (mouse). (TIF) [file pone.0050300.s002.tif]

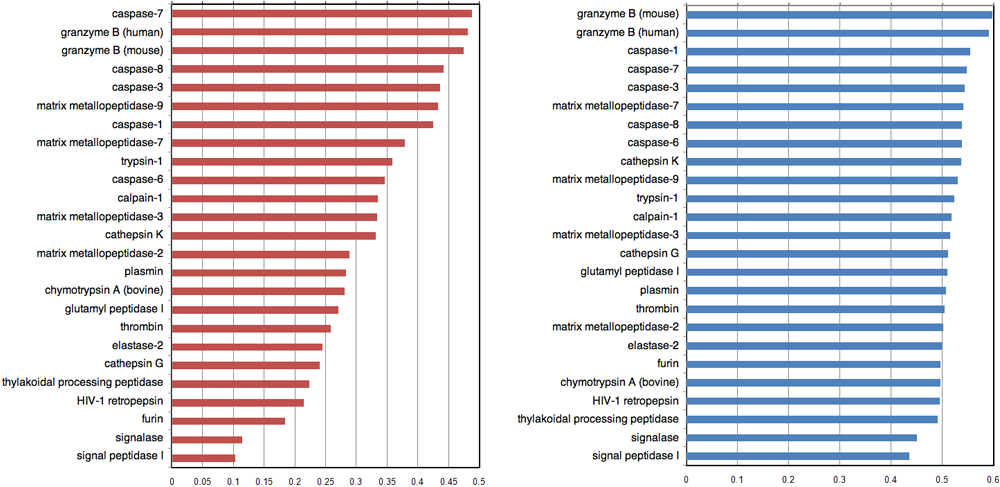

Supplement: Figure S3 — Enrichment analysis of natively disordered residues and solvent exposed residues across different protease substrate types. Left: protease substrate categories that are enriched in natively disordered residues; Right: protease substrate categories that are enriched in solvent exposed residues. Higher percentage on the x-axis indicates greater enrichment of either native disorder or solvent accessibility. (TIF) [file pone.0050300.s003.tif]

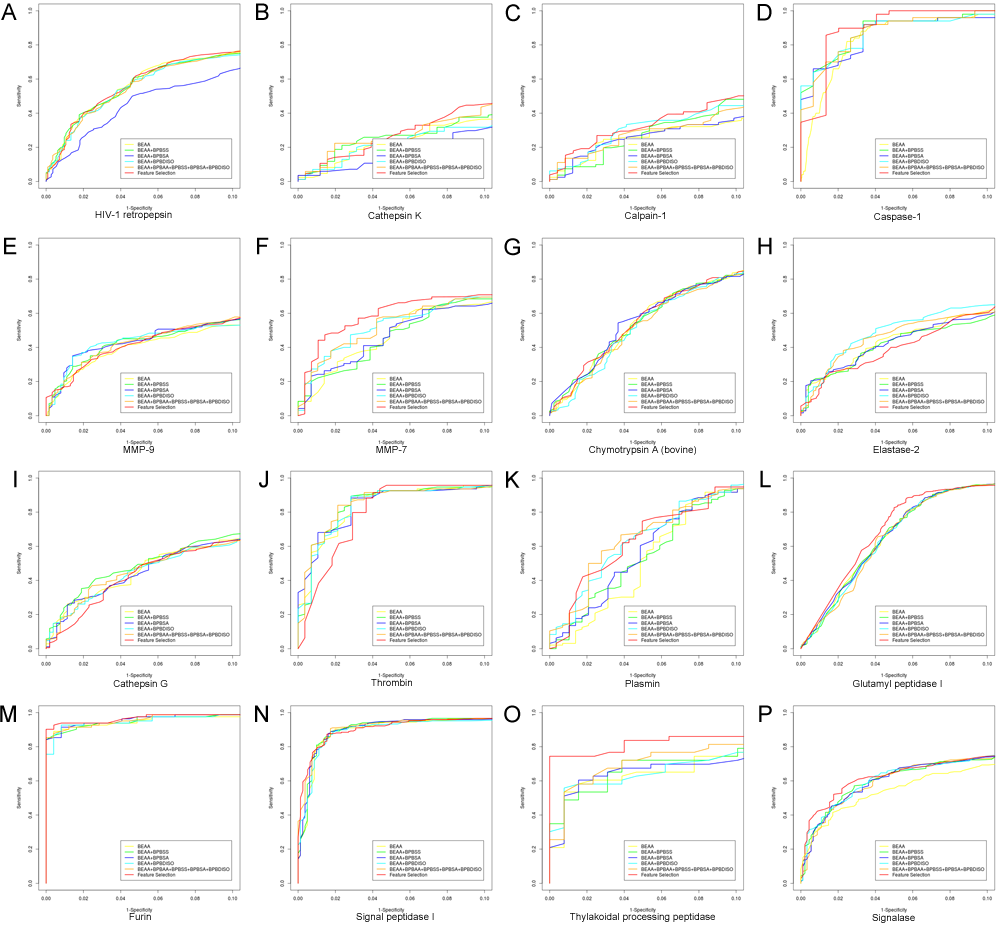

Supplement: Figure S4 — Assessing the performance of PROSPER models for cleavage site prediction of the 16 proteases, based on gradually increased features to evaluate the relative contribution of each type of feature. Panels A–P correspond to: A, HIV-1 retropepsin; B, cathepsin K; C, calpain-1; D, MMP-9; E, MMP-3; F, MMP-7; G, chymotrypsin A (bovine); H, elastase-2; I, cathepsin G; J, thrombin; K, plasmin; L, glutamyl peptidase I; M, furin; N, signal peptidase I; O, thylakoidal processing peptidase; and P, signalase, which are presented according to the alphabetical order of their MEROPS ID in Table 1. For clarity, the ROC curves with high prediction specificities (90–100%) were displayed. (TIF) [file pone.0050300.s004.tif]

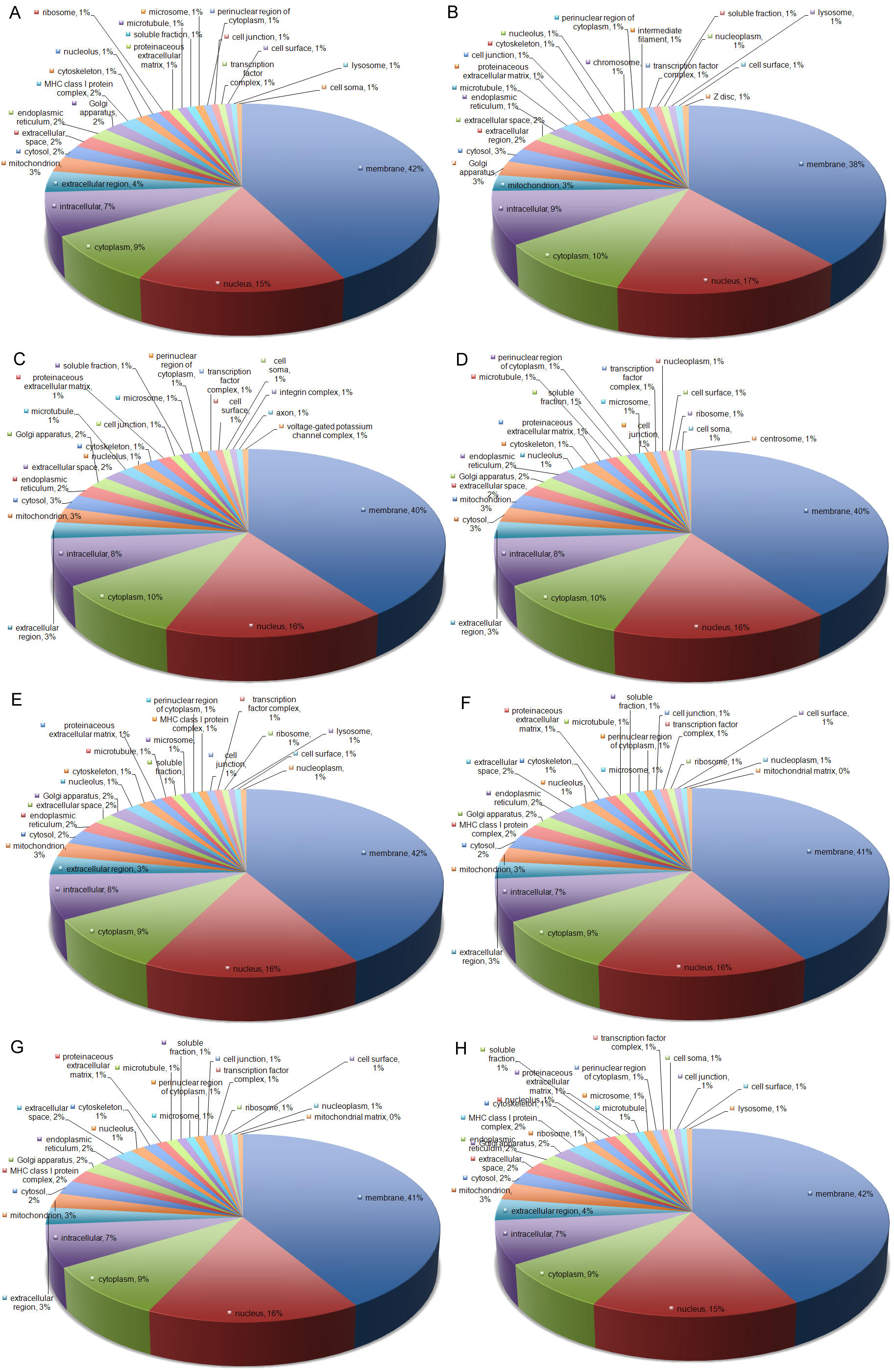

Supplement: Figure S5 — Distribution of the Gene Ontology annotations of the predicted protease substrates. A) caspase-1; B) caspase-3; C) caspase-7, D) caspase-6, E) caspase-8, F) granzyme B (human), G) granzyme B (mouse), and H) the background distribution based on the whole human proteome. (TIF) [file pone.0050300.s005.tif]
