# Supplementary material for: PROSPER: An Integrated Feature-Based Tool for Predicting Protease Substrate Cleavage Sites
Source: PLoS One. 2012 Nov 29;7(11):e50300. doi: 10.1371/journal.pone.0050300 (PMC3510211; doi:10.1371/journal.pone.0050300)
Supplement: Table S1 — Predictive performance based on singe sequence inputs only (sequence encoding scheme “BEAA”), with the local window size of P4-P2′. The results were obtained by 5-fold cross-validation tests. (DOC) [file pone.0050300.s006.doc]

**Table S1**. Predictive performance based on single sequence inputs only (sequence encoding scheme “BEAA”), with a local window size of P4-P2′. Results were obtained by 5-fold cross-validation tests.

| **Protease family** | **Protease** | **Merops ID** | **Accuracy**  **(%)** | **Sensitivity**  **(%)** | **Specificity**  **(%)** | **F-score**  **(%)** | **MCC** |
| --- | --- | --- | --- | --- | --- | --- | --- |
| **Aspartic protease** | HIV-1 retropepsin | A02.001 | 85.6 | 53.7 | 96.2 | 65.1 | 0.627 |
| **Cysteine protease** | Cathepsin K | C01.036 | 77.6 | 45.9 | 88.2 | 50.6 | 0.504 |
|  | Calpain-1 | C02.001 | 78.7 | 22.2 | 97.5 | 34.3 | 0.390 |
|  | Caspase-1 | C14.001 | 83.5 | 34.0 | 100 | 50.7 | 0.528 |
|  | Caspase-3 | C14.003 | 91.6 | 69.1 | 99.1 | 80.5 | 0.776 |
|  | Caspase-7 | C14.004 | 85.7 | 43.8 | 99.6 | 60.5 | 0.601 |
|  | Caspase-6 | C14.005 | 88.8 | 55.7 | 99.8 | 71.3 | 0.693 |
|  | Caspase-8 | C14.009 | 84.9 | 41.4 | 99.4 | 57.8 | 0.579 |
| **Metalloprotease** | Matrix metallopeptidase-2 | M10.003 | 86.7 | 80.6 | 88.7 | 75.2 | 0.706 |
|  | Matrix metallopeptidase-9 | M10.004 | 80.2 | 23.7 | 99.1 | 37.5 | 0.421 |
|  | Matrix metallopeptidase-3 | M10.005 | 78.6 | 20.4 | 98.0 | 32.3 | 0.377 |
|  | Matrix metallopeptidase-7 | M10.008 | 80.3 | 27.4 | 97.9 | 40.9 | 0.442 |
| **Serine protease** | Chymotrypsin A (bovine) | S01.001 | 88.5 | 84.0 | 90.0 | 78.5 | 0.740 |
|  | Granzyme B (human) | S01.010 | 97.1 | 96.4 | 97.3 | 94.3 | 0.926 |
|  | Elastase-2 | S01.131 | 80.7 | 31.3 | 97.2 | 44.8 | 0.469 |
|  | Cathepsin G | S01.133 | 80.5 | 72.7 | 83.1 | 65.1 | 0.610 |
|  | Granzyme B (mouse) | S01.136 | 89.1 | 59.7 | 98.9 | 73.3 | 0.708 |
|  | Thrombin | S01.217 | 84.8 | 41.5 | 99.3 | 57.8 | 0.578 |
|  | Plasmin | S01.233 | 83.6 | 49.0 | 95.1 | 59.9 | 0.581 |
|  | Glutamyl peptidase I | S01.269 | 91.1 | 82.1 | 94.1 | 82.2 | 0.784 |
|  | Furin | S08.071 | 86.9 | 47.6 | 100 | 64.5 | 0.636 |
|  | Signal peptidase I | S26.001 | 92.7 | 73.2 | 99.1 | 83.3 | 0.805 |
|  | Thylakoidal processing peptidase | S26.008 | 88.4 | 60.5 | 97.7 | 72.2 | 0.694 |
|  | Signalase | S26.010 | 85.1 | 47.5 | 97.7 | 61.5 | 0.602 |
