# Supplementary material for: PROSPER: An Integrated Feature-Based Tool for Predicting Protease Substrate Cleavage Sites
Source: PLoS One. 2012 Nov 29;7(11):e50300. doi: 10.1371/journal.pone.0050300 (PMC3510211; doi:10.1371/journal.pone.0050300)
Supplement: Table S2 — Predictive performance based on singe sequence inputs only (sequence encoding scheme “BEAA+BPBSS”), with the local window size of P4-P2′. The results were obtained by 5-fold cross-validation tests. (DOC) [file pone.0050300.s007.doc]

**Table S2**. Predictive performance based on single sequence inputs only (sequence encoding scheme “BEAA+BPBSS”), with a local window size of P4-P2′. Results were obtained by 5-fold cross-validation tests.

| **Protease family** | **Protease** | **Merops ID** | **Accuracy**  **(%)** | **Sensitivity**  **(%)** | **Specificity**  **(%)** | **F-score**  **(%)** | **MCC** |
| --- | --- | --- | --- | --- | --- | --- | --- |
| **Aspartic protease** | HIV-1 retropepsin | A02.001 | 85.2 | 80.1 | 86.9 | 73.0 | 0.683 |
| **Cysteine protease** | Cathepsin K | C01.036 | 73.2 | 55.3 | 79.2 | 50.8 | 0.505 |
|  | Calpain-1 | C02.001 | 74.7 | 61.7 | 79.0 | 54.9 | 0.531 |
|  | Caspase-1 | C14.001 | 86.0 | 44.0 | 100 | 61.1 | 0.609 |
|  | Caspase-3 | C14.003 | 93.4 | 77.0 | 98.9 | 85.4 | 0.826 |
|  | Caspase-7 | C14.004 | 86.2 | 47.2 | 99.3 | 93.2 | 0.621 |
|  | Caspase-6 | C14.005 | 91.8 | 68.9 | 99.4 | 80.7 | 0.779 |
|  | Caspase-8 | C14.009 | 87.1 | 48.3 | 100 | 65.1 | 0.642 |
| **Metalloprotease** | Matrix metallopeptidase-2 | M10.003 | 86.7 | 60.3 | 95.5 | 69.3 | 0.662 |
|  | Matrix metallopeptidase-9 | M10.004 | 80.8 | 27.5 | 98.6 | 71.7 | 0.451 |
|  | Matrix metallopeptidase-3 | M10.005 | 78.5 | 17.8 | 98.7 | 29.2 | 0.356 |
|  | Matrix metallopeptidase-7 | M10.008 | 79.7 | 22.1 | 98.9 | 35.3 | 0.404 |
| **Serine protease** | Chymotrypsin A (bovine) | S01.001 | 85.1 | 53.9 | 95.4 | 64.4 | 0.619 |
|  | Granzyme B (human) | S01.010 | 94.2 | 80.4 | 98.8 | 87.4 | 0.847 |
|  | Elastase-2 | S01.131 | 80.6 | 72.3 | 83.4 | 65.1 | 0.610 |
|  | Cathepsin G | S01.133 | 81.3 | 30.1 | 98.3 | 44.5 | 0.470 |
|  | Granzyme B (mouse) | S01.136 | 91.6 | 73.4 | 97.6 | 81.3 | 0.780 |
|  | Thrombin | S01.217 | 88.3 | 55.3 | 99.3 | 70.3 | 0.682 |
|  | Plasmin | S01.233 | 84.1 | 52.1 | 94.8 | 62.1 | 0.599 |
|  | Glutamyl peptidase I | S01.269 | 91.4 | 85.1 | 93.6 | 83.2 | 0.794 |
|  | Furin | S08.071 | 86.3 | 45.1 | 100 | 62.2 | 0.618 |
|  | Signal peptidase I | S26.001 | 94.2 | 79.9 | 99.0 | 87.4 | 0.847 |
|  | Thylakoidal processing peptidase | S26.008 | 78.5 | 14.0 | 100 | 24.5 | 0.329 |
|  | Signalase | S26.010 | 85.7 | 49.5 | 97.8 | 64.2 | 0.618 |
