# Supplementary material for: PROSPER: An Integrated Feature-Based Tool for Predicting Protease Substrate Cleavage Sites
Source: PLoS One. 2012 Nov 29;7(11):e50300. doi: 10.1371/journal.pone.0050300 (PMC3510211; doi:10.1371/journal.pone.0050300)
Supplement: Table S3 — Predictive performance based on singe sequence inputs only (sequence encoding scheme “BEAA+BPBSA”), with the local window size of P4-P2′. The results were obtained by 5-fold cross-validation tests. (DOC) [file pone.0050300.s008.doc]

**Table S3**. Predictive performance based on single sequence inputs only (sequence encoding scheme “BEAA+BPBSA”), with a local window size of P4-P2′. Results were obtained by 5-fold cross-validation tests.

| **Protease family** | **Protease** | **Merops ID** | **Accuracy**  **(%)** | **Sensitivity**  **(%)** | **Specificity**  **(%)** | **F-score**  **(%)** | **MCC** |
| --- | --- | --- | --- | --- | --- | --- | --- |
| **Aspartic protease** | HIV-1 retropepsin | A02.001 | 85.6 | 55.9 | 95.6 | 66.0 | 0.634 |
| **Cysteine protease** | Cathepsin K | C01.036 | 79.4 | 43.5 | 91.4 | 51.4 | 0.510 |
|  | Calpain-1 | C02.001 | 78.4 | 22.2 | 97.1 | 34.0 | 0.387 |
|  | Caspase-1 | C14.001 | 84.5 | 38.0 | 100 | 55.1 | 0.561 |
|  | Caspase-3 | C14.003 | 94.0 | 79.9 | 98.7 | 87.0 | 0.842 |
|  | Caspase-7 | C14.004 | 88.8 | 56.2 | 99.6 | 71.4 | 0.694 |
|  | Caspase-6 | C14.005 | 89.7 | 59.9 | 99.6 | 74.3 | 0.720 |
|  | Caspase-8 | C14.009 | 85.8 | 44.8 | 99.4 | 61.2 | 0.606 |
| **Metalloprotease** | Matrix metallopeptidase-2 | M10.003 | 86.7 | 59.2 | 95.9 | 69.0 | 0.660 |
|  | Matrix metallopeptidase-9 | M10.004 | 80.9 | 27.0 | 98.9 | 41.5 | 0.450 |
|  | Matrix metallopeptidase-3 | M10.005 | 78.8 | 22.4 | 97.6 | 34.5 | 0.392 |
|  | Matrix metallopeptidase-7 | M10.008 | 80.8 | 30.5 | 97.5 | 44.3 | 0.466 |
| **Serine protease** | Chymotrypsin A (bovine) | S01.001 | 88.8 | 87.4 | 89.3 | 79.6 | 0.751 |
|  | Granzyme B (human) | S01.010 | 97.3 | 96.4 | 97.6 | 94.7 | 0.930 |
|  | Elastase-2 | S01.131 | 81.1 | 72.3 | 84.1 | 65.7 | 0.616 |
|  | Cathepsin G | S01.133 | 81.1 | 34.1 | 96.8 | 47.4 | 0.487 |
|  | Granzyme B (mouse) | S01.136 | 92.9 | 79.9 | 97.2 | 84.8 | 0.816 |
|  | Thrombin | S01.217 | 88.7 | 57.4 | 98.9 | 71.5 | 0.692 |
|  | Plasmin | S01.233 | 84.1 | 51.0 | 95.1 | 61.6 | 0.596 |
|  | Glutamyl peptidase I | S01.269 | 91.0 | 81.8 | 94.1 | 82.0 | 0.782 |
|  | Furin | S08.071 | 91.2 | 64.6 | 100 | 78.6 | 0.760 |
|  | Signal peptidase I | S26.001 | 93.5 | 76.9 | 99.0 | 85.5 | 0.828 |
|  | Thylakoidal processing peptidase | S26.008 | 89.0 | 60.5 | 98.5 | 73.2 | 0.705 |
|  | Signalase | S26.010 | 85.5 | 48.8 | 97.7 | 62.7 | 0.611 |
