# Supplementary material for: PROSPER: An Integrated Feature-Based Tool for Predicting Protease Substrate Cleavage Sites
Source: PLoS One. 2012 Nov 29;7(11):e50300. doi: 10.1371/journal.pone.0050300 (PMC3510211; doi:10.1371/journal.pone.0050300)
Supplement: Table S4 — Predictive performance based on singe sequence inputs only (sequence encoding “BEAA+BPBDISO”), with the local window size of P4-P2′. The results were obtained by 5-fold cross-validation tests. (DOC) [file pone.0050300.s009.doc]

**Table S4**. Predictive performance based on single sequence inputs only (sequence encoding “BEAA+BPBDISO”), with a local window size of P4-P2′. Results were obtained by 5-fold cross-validation tests.

| **Protease family** | **Protease** | **Merops ID** | **Accuracy**  **(%)** | **Sensitivity**  **(%)** | **Specificity**  **(%)** | **F-score**  **(%)** | **MCC** |
| --- | --- | --- | --- | --- | --- | --- | --- |
| **Aspartic protease** | HIV-1 retropepsin | A02.001 | 85.7 | 56.4 | 95.5 | 66.4 | 0.636 |
| **Cysteine protease** | Cathepsin K | C01.036 | 77.6 | 20.0 | 96.9 | 30.9 | 0.363 |
|  | Calpain-1 | C02.001 | 80.2 | 33.3 | 95.9 | 45.8 | 0.472 |
|  | Caspase-1 | C14.001 | 86.0 | 44.0 | 100 | 61.1 | 0.609 |
|  | Caspase-3 | C14.003 | 94.0 | 79.9 | 98.7 | 87.0 | 0.842 |
|  | Caspase-7 | C14.004 | 86.8 | 48.3 | 99.6 | 64.7 | 0.636 |
|  | Caspase-6 | C14.005 | 92.4 | 70.7 | 99.6 | 82.2 | 0.795 |
|  | Caspase-8 | C14.009 | 87.5 | 53.4 | 98.9 | 68.1 | 0.612 |
| **Metalloprotease** | Matrix metallopeptidase-2 | M10.003 | 86.4 | 79.4 | 88.7 | 74.4 | 0.699 |
|  | Matrix metallopeptidase-9 | M10.004 | 81.5 | 30.3 | 98.6 | 75.1 | 0.476 |
|  | Matrix metallopeptidase-3 | M10.005 | 78.8 | 20.3 | 98.2 | 32.5 | 0.379 |
|  | Matrix metallopeptidase-7 | M10.008 | 81.1 | 27.4 | 98.9 | 41.9 | 0.454 |
| **Serine protease** | Chymotrypsin A (bovine) | S01.001 | 84.5 | 87.0 | 89.0 | 79.1 | 0.745 |
|  | Granzyme B (human) | S01.010 | 97.0 | 96.4 | 97.2 | 94.2 | 0.924 |
|  | Elastase-2 | S01.131 | 82.7 | 38.2 | 97.6 | 52.5 | 0.529 |
|  | Cathepsin G | S01.133 | 81.0 | 31.8 | 97.3 | 45.5 | 0.474 |
|  | Granzyme B (mouse) | S01.136 | 91.2 | 70.1 | 98.3 | 80.0 | 0.769 |
|  | Thrombin | S01.217 | 90.4 | 66.0 | 98.6 | 77.5 | 0.746 |
|  | Plasmin | S01.233 | 85.9 | 53.1 | 96.9 | 65.4 | 0.631 |
|  | Glutamyl peptidase I | S01.269 | 91.4 | 84.6 | 93.7 | 83.2 | 0.794 |
|  | Furin | S08.071 | 92.7 | 70.7 | 100 | 82.9 | 0.803 |
|  | Signal peptidase I | S26.001 | 94.0 | 79.2 | 98.9 | 86.8 | 0.840 |
|  | Thylakoidal processing peptidase | S26.008 | 88.4 | 55.8 | 99.2 | 70.6 | 0.685 |
|  | Signalase | S26.010 | 85.8 | 75.2 | 89.3 | 72.6 | 0.683 |
