# Supplementary material for: PROSPER: An Integrated Feature-Based Tool for Predicting Protease Substrate Cleavage Sites
Source: PLoS One. 2012 Nov 29;7(11):e50300. doi: 10.1371/journal.pone.0050300 (PMC3510211; doi:10.1371/journal.pone.0050300)
Supplement: Table S5 — The AUC (area under ROC curve) values for PROSPER models based on different sequence encoding schemes: “BEAA”, “BEAA+BPBAA+BPBSS+BPBSA+BPBDISO” (termed as “ALL” here) without feature selection, and “BEAA+BPBAA+BPBSS+BPBSA+BPBDISO with feature selection. An extended local window of P8-P8′ was used to build the PROSPER models. See the main text for details of different sequence encoding schemes. (DOC) [file pone.0050300.s010.doc]

**Table S5**. The AUC (area under ROC curve) values for PROSPER models based on different sequence encoding schemes: “BEAA”, “BEAA+BPBAA+BPBSS+BPBSA+BPBDISO” (termed as “ALL”) without feature selection, and “BEAA+BPBAA+BPBSS+BPBSA+BPBDISO with feature selection. An extended local window of P8-P8′ was used to build the PROSPER models. See the main text for details of different sequence encoding schemes.

| **Protease family** | **Protease** | **Merops ID** | **BEAA**^a^ | | **ALL**^b^ | | **ALL_select**^c^ | |
| --- | --- | --- | --- | --- | --- | --- | --- | --- |
|  |  |  | AUC | Number of features | AUC | Number of features | AUC | Number of features |
| **Aspartic protease** | HIV-1 retropepsin | A02.001 | 0.879 | 320 | 0.875 | 448 | 0.850 | 32 |
| **Cysteine protease** | Cathepsin K | C01.036 | 0.682 | 320 | 0.690 | 448 | 0.695 | 34 |
|  | Calpain-1 | C02.001 | 0.634 | 320 | 0.678 | 448 | 0.715 | 37 |
|  | Caspase-1 | C14.001 | 0.850 | 320 | 0.917 | 448 | 0.902 | 18 |
|  | Caspase-3 | C14.003 | 0.953 | 320 | 0.966 | 448 | 0.982 | 9 |
|  | Caspase-7 | C14.004 | 0.918 | 320 | 0.915 | 448 | 0.929 | 9 |
|  | Caspase-6 | C14.005 | 0.971 | 320 | 0.972 | 448 | 0.964 | 9 |
|  | Caspase-8 | C14.009 | 0.926 | 320 | 0.938 | 448 | 0.933 | 9 |
| **Metalloprotease** | Matrix metallopeptidase-2 | M10.003 | 0.906 | 320 | 0.904 | 448 | 0.889 | 24 |
|  | Matrix metallopeptidase-9 | M10.004 | 0.763 | 320 | 0.758 | 448 | 0.754 | 37 |
|  | Matrix metallopeptidase-3 | M10.005 | 0.769 | 320 | 0.759 | 448 | 0.738 | 35 |
|  | Matrix metallopeptidase-7 | M10.008 | 0.770 | 320 | 0.791 | 448 | 0.804 | 22 |
| **Serine protease** | Chymotrypsin A (bovine) | S01.001 | 0.890 | 320 | 0.883 | 448 | 0.893 | 14 |
|  | Granzyme B (human) | S01.010 | 0.969 | 320 | 0.956 | 448 | 0.947 | 6 |
|  | Elastase-2 | S01.131 | 0.824 | 320 | 0.825 | 448 | 0.813 | 37 |
|  | Cathepsin G | S01.133 | 0.792 | 320 | 0.793 | 448 | 0.801 | 36 |
|  | Granzyme B (mouse) | S01.136 | 0.956 | 320 | 0.958 | 448 | 0.962 | 7 |
|  | Thrombin | S01.217 | 0.903 | 320 | 0.883 | 448 | 0.828 | 6 |
|  | Plasmin | S01.233 | 0.810 | 320 | 0.829 | 448 | 0.853 | 13 |
|  | Glutamyl peptidase I | S01.269 | 0.932 | 320 | 0.931 | 448 | 0.914 | 3 |
|  | Furin | S08.071 | 0.906 | 320 | 0.916 | 448 | 0.911 | 10 |
|  | Signal peptidase I | S26.001 | 0.932 | 320 | 0.945 | 448 | 0.903 | 16 |
|  | Thylakoidal processing peptidase | S26.008 | 0.694 | 320 | 0.716 | 448 | 0.734 | 22 |
|  | Signalase | S26.010 | 0.850 | 320 | 0.860 | 448 | 0.866 | 28 |

^a^sequence encoding scheme “BEAA”;

^b^sequence encoding scheme “BEAA+BPBAA+BPBSS+BPBSA+BPBDISO” without feature selection;

^c^sequence encoding scheme “BEAA+BPBAA+BPBSS+BPBSA+BPBDISO” with feature selection.
