# Supplementary material for: PROSPER: An Integrated Feature-Based Tool for Predicting Protease Substrate Cleavage Sites
Source: PLoS One. 2012 Nov 29;7(11):e50300. doi: 10.1371/journal.pone.0050300 (PMC3510211; doi:10.1371/journal.pone.0050300)
Supplement: Table S6 — List of the more informative features selected using random forest algorithm. Features with a Z score greater than 1.0 are selected and considered to be more informative. An extended local window size of P8-P8′ was used to perform feature selection in order to extract more relevant features. (DOC) [file pone.0050300.s011.doc]

**Table S6**. List of the more informative features selected using random forest algorithm. Features with a Z score greater than 1.0 were selected and considered to be more informative. An extended local window size of P8-P8′ using the sequence encoding scheme “ALL” was used to perform feature selection in order to extract more relevant features. The order and dimensionality for each type of sequence profile can be found in Table S7.

| **Protease family** | **Protease** | **Merops ID** | **List of the positions of selected features** |
| --- | --- | --- | --- |
| **Aspartic protease** | HIV-1 retropepsin | A02.001 | 136,196,321,322,323,324,325,326,327,328,329,330,331,332,333,334,335,336,338,339,340,341,342,343,344,345,346,347,348,349,351,377 |
| **Cysteine protease** | Cathepsin K | C01.036 | 122,127,176,275,322,323,324,325,326,327,328,329,330,331,332,333,334,335,336,337,339,340,341,342,343,344,345,346,347,348,349,350,351,352 |
|  | Calpain-1 | C02.001 | 122,169,207,321,322,324,325,326,327,328,329,330,331,332,334,335,336,337,338,339,340,341,342,343,344,345,346,347,348,349,350,351,352,427,429,443,445 |
|  | Caspase-1 | C14.001 | 95,155,321,323,325,326,327,328,329,330,331,332,333,341,343,344,346,348 |
|  | Caspase-3 | C14.003 | 95,155,325,326,327,328,329,341,344 |
|  | Caspase-7 | C14.004 | 95,155,180,325,327,328,329,330,344 |
|  | Caspase-6 | C14.005 | 85,116,155,325,326,328,341,342,344 |
|  | Caspase-8 | C14.009 | 116,155,325,326,327,328,329,332,344 |
| **Metalloprotease** | Matrix metallopeptidase-2 | M10.003 | 107,162,163,322,326,327,328,329,330,331,332,333,334,335,336,342,343,344,345,346,347,349,393,409 |
|  | Matrix metallopeptidase-9 | M10.004 | 40,107,162,220,280,321,322,323,324,325,326,327,328,329,330,331,332,333,334,335,336,337,338,339,340,341,342,343,344,345,346,347,348,349,350,351,352 |
|  | Matrix metallopeptidase-3 | M10.005 | 107,129,162,321,322,323,324,325,326,327,328,329,330,331,332,333,334,335,336,337,338,339,340,341,342,343,344,345,346,347,348,349,350,351,352 |
|  | Matrix metallopeptidase-7 | M10.008 | 107,156,162,321,324,325,326,327,328,329,330,331,332,334,335,337,339,342,343,344,345,349 |
| **Serine protease** | Chymotrypsin A (bovine) | S01.001 | 141,142,150,321,322,323,325,326,327,328,329,344,392,408 |
|  | Granzyme B (human) | S01.010 | 155,325,327,328,330,344 |
|  | Elastase-2 | S01.131 | 127,143,145,182,227,321,322,323,324,325,326,327,328,329,330,331,332,333,334,335,336,337,338,339,340,341,342,343,344,345,346,347,348,349,350,351,352 |
|  | Cathepsin G | S01.133 | 141,142,321,322,323,324,325,326,327,328,329,330,331,332,333,334,335,336,337,338,339,340,341,342,343,344,345,346,347,348,349,350,351,352,392,408 |
|  | Granzyme B (mouse) | S01.136 | 155,325,327,328,330,341,344 |
|  | Thrombin | S01.217 | 159,327,328,329,331,344 |
|  | Plasmin | S01.233 | 154,159,322,324,325,328,329,330,332,333,340,344,346 |
|  | Glutamyl peptidase I | S01.269 | 156,328,344 |
|  | Furin | S08.071 | 99,134,159,325,326,327,328,329,341,344 |
|  | Signal peptidase I | S26.001 | 109,149,321,322,323,326,327,328,329,330,339,342,344,353,354,386 |
|  | Thylakoidal processing peptidase | S26.008 | 109,149,321,322,323,325,326,327,328,329,330,331,332,334,336,338,342,343,344,352,366,382 |
|  | Signalase | S26.010 | 105,149,321,322,323,324,326,327,328,329,330,331,332,337,342,343,344,353,354,355,369,370,385,386,387,401,402,403 |
