# Supplementary material for: PROSPER: An Integrated Feature-Based Tool for Predicting Protease Substrate Cleavage Sites
Source: PLoS One. 2012 Nov 29;7(11):e50300. doi: 10.1371/journal.pone.0050300 (PMC3510211; doi:10.1371/journal.pone.0050300)
Supplement: Table S7 — The numbering and categorization of all feature vectors in the encoding scheme “ALL”. An extended local window size of P8-P8′ using the sequence encoding scheme “ALL” was used to perform feature selection in order to extract more relevant features. (DOC) [file pone.0050300.s012.doc]

**Table S7**. The numbering and categorization of all feature vectors in the encoding scheme “ALL”. An extended local window size of P8-P8′ using the sequence encoding scheme “ALL” was used to perform feature selection in order to extract more relevant features.

| **Order^a^** | **Dimensionality** | **Sequence profile** | **Abbreviation** |
| --- | --- | --- | --- |
| **1-320** | 320 | binary encoding amino acid profile | BEAA |
| **321-352** | 32 | bi-profile Bayesian amino acid profile | BPBAA |
| **353-384** | 32 | bi-profile Bayesian secondary structure profile | BPBSS |
| **385-416** | 32 | bi-profile Bayesian solvent accessibility profile | BPBSA |
| **417-448** | 32 | bi-profile Bayesian disordered profile | BPBDISO |

^a^ The order of feature vector among the total of 168 features for the sequence encoding scheme ‘ALL’ based on the local window size of P8-P8′.
