# Supplementary material for: PROSPER: An Integrated Feature-Based Tool for Predicting Protease Substrate Cleavage Sites
Source: PLoS One. 2012 Nov 29;7(11):e50300. doi: 10.1371/journal.pone.0050300 (PMC3510211; doi:10.1371/journal.pone.0050300)
Supplement: Table S8 — The significantly enriched Gene Ontology (GO) terms of the predicted substrates of caspase-1, 7, 6, 8, granzyme B (human) and granzyme B (mouse) that were available to be analyzed by the gene list enrichment analysis tool ToppFun. The significantly enriched GO terms of the predicted substrates are listed according to three major categories: Molecular Function, Biological Process and Cellular Component. The P-value of each GO term in the predicted substrates was calculated by randomly sampling the whole genome. (DOC) [file pone.0050300.s013.doc]

**Table S8**. The significantly enriched Gene Ontology (GO) terms of the predicted substrates of caspase-1, 7, 6, 8, granzyme B (human) and granzyme B (mouse) that were available to be analyzed by the gene list enrichment analysis tool ToppFun. The significantly enriched GO terms of the predicted substrates are listed according to three major categories: Molecular Function, Biological Process and Cellular Component. The *P*-value of each GO term in the predicted substrates was calculated by randomly sampling the whole genome.

| **Protease** | **Gene Ontology category** | **Rank** | **ID** | **Name** | **P-value** | **Term in predicted substrates** | **Term in human Genome** |
| --- | --- | --- | --- | --- | --- | --- | --- |
| Caspase-1 | Molecular Function | 1 | GO:0016772 | transferase activity, transferring phosphorus-containing groups | 3.265E-16 | 954 | 984 |
|  |  | 2 | GO:0016301 | kinase activity | 1.375E-15 | 831 | 854 |
|  |  | 3 | GO:0016773 | phosphotransferase activity, alcohol group as acceptor | 2.694E-13 | 709 | 728 |
|  |  | 4 | GO:0019899 | enzyme binding | 5.377E-13 | 808 | 834 |
|  |  | 5 | GO:0046983 | protein dimerization activity | 1.293E-11 | 726 | 749 |
|  |  | 6 | GO:0030528 | transcription regulator activity | 1.856E-11 | 1012 | 1056 |
|  |  | 7 | GO:0042802 | identical protein binding | 2.701E-11 | 804 | 833 |
|  |  | 8 | GO:0019904 | protein domain specific binding | 2.273E-10 | 504 | 515 |
|  |  | 9 | GO:0004672 | protein kinase activity | 3.695E-10 | 596 | 613 |
|  |  | 10 | GO:0001071 | nucleic acid binding transcription factor activity | 4.660E-10 | 968 | 1012 |
|  | Biological Process | 1 | GO:0007049 | cell cycle | 4.198E-23 | 1214 | 1248 |
|  |  | 2 | GO:0010604 | positive regulation of macromolecule metabolic process | 8.815E-22 | 1300 | 1342 |
|  |  | 3 | GO:0048468 | cell development | 1.204E-21 | 1207 | 1243 |
|  |  | 4 | GO:0009893 | positive regulation of metabolic process | 1.350E-21 | 1410 | 1460 |
|  |  | 5 | GO:0051239 | regulation of multicellular organismal process | 2.418E-21 | 1403 | 1453 |
|  |  | 6 | GO:0031325 | positive regulation of cellular metabolic process | 5.120E-20 | 1338 | 1386 |
|  |  | 7 | GO:0010646 | regulation of cell communication | 1.197E-17 | 1071 | 1105 |
|  |  | 8 | GO:0030030 | cell projection organization | 1.099E-16 | 760 | 776 |
|  |  | 9 | GO:0050793 | regulation of developmental process | 1.340E-16 | 983 | 1013 |
|  |  | 10 | GO:0031324 | negative regulation of cellular metabolic process | 2.250E-16 | 992 | 1023 |
|  | Cellular Component | 1 | GO:0005654 | Nucleoplasm | 3.358E-26 | 1299 | 1341 |
|  |  | 2 | GO:0031226 | intrinsic to plasma membrane | 2.638E-19 | 1269 | 1322 |
|  |  | 3 | GO:0005730 | nucleolus | 4.608E-19 | 1093 | 1133 |
|  |  | 4 | GO:0005887 | integral to plasma membrane | 2.365E-18 | 1243 | 1296 |
|  |  | 5 | GO:0044451 | nucleoplasm part | 6.754E-18 | 763 | 782 |
|  |  | 6 | GO:0005794 | Golgi apparatus | 3.068E-15 | 992 | 1032 |
|  |  | 7 | GO:0042995 | cell projection | 1.403E-14 | 1025 | 1069 |
|  |  | 8 | GO:0031988 | membrane-bounded vesicle | 2.378E-11 | 743 | 772 |
|  |  | 9 | GO:0016023 | cytoplasmic membrane-bounded vesicle | 4.624E-11 | 722 | 750 |
|  |  | 10 | GO:0000267 | cell fraction | 5.254E-11 | 1378 | 1462 |
| Caspase-7 | Molecular Function | 1 | GO:0019899 | enzyme binding | 1.269E-15 | 317 | 834 |
|  |  | 2 | GO:0005083 | small GTPase regulator activity | 3.316E-13 | 141 | 307 |
|  |  | 3 | GO:0030695 | GTPase regulator activity | 9.582E-13 | 192 | 464 |
|  |  | 4 | GO:0060589 | nucleoside-triphosphatase regulator activity | 1.166E-11 | 193 | 477 |
|  |  | 5 | GO:0008092 | cytoskeletal protein binding | 5.035E-11 | 223 | 579 |
|  |  | 6 | GO:0005085 | guanyl-nucleotide exchange factor activity | 1.546E-10 | 83 | 160 |
|  |  | 7 | GO:0017111 | nucleoside-triphosphatase activity | 7.398E-10 | 276 | 768 |
|  |  | 8 | GO:0016462 | pyrophosphatase activity | 4.463E-9 | 282 | 799 |
|  |  | 9 | GO:0016818 | hydrolase activity, acting on acid anhydrides, in phosphorus-containing anhydrides | 7.251E-9 | 282 | 802 |
|  |  | 10 | GO:0016817 | hydrolase activity, acting on acid anhydrides | 9.987E-9 | 282 | 804 |
|  | Biological Process | 1 | GO:0030030 | cell projection organization | 3.239E-17 | 305 | 776 |
|  |  | 2 | GO:0022008 | neurogenesis | 1.339E-16 | 376 | 1015 |
|  |  | 3 | GO:0048699 | generation of neurons | 2.786E-16 | 358 | 959 |
|  |  | 4 | GO:0032990 | cell part morphogenesis | 6.364E-16 | 236 | 570 |
|  |  | 5 | GO:0048858 | cell projection morphogenesis | 6.728E-16 | 234 | 564 |
|  |  | 6 | GO:0048666 | neuron development | 1.273E-15 | 281 | 715 |
|  |  | 7 | GO:0048468 | cell development | 2.114E-15 | 439 | 1243 |
|  |  | 8 | GO:0030182 | neuron differentiation | 2.508E-15 | 335 | 894 |
|  |  | 9 | GO:0031175 | neuron projection development | 1.022E-14 | 250 | 625 |
|  |  | 10 | GO:0048812 | neuron projection morphogenesis | 7.651E-14 | 215 | 523 |
|  | Cellular Component | 1 | GO:0044430 | cytoskeletal part | 1.304E-14 | 393 | 1120 |
|  |  | 2 | GO:0015630 | microtubule cytoskeleton | 4.588E-14 | 263 | 689 |
|  |  | 3 | GO:0044451 | nucleoplasm part | 5.182E-11 | 280 | 782 |
|  |  | 4 | GO:0042995 | cell projection | 5.616E-11 | 363 | 1069 |
|  |  | 5 | GO:0005654 | nucleoplasm | 8.288E-11 | 439 | 1341 |
|  |  | 6 | GO:0030054 | cell junction | 2.539E-9 | 228 | 628 |
|  |  | 7 | GO:0043005 | neuron projection | 2.337E-8 | 211 | 583 |
|  |  | 8 | GO:0045202 | synapse | 7.087E-8 | 176 | 472 |
|  |  | 9 | GO:0044463 | cell projection part | 7.723E-8 | 187 | 509 |
|  |  | 10 | GO:0070161 | anchoring junction | 3.189E-7 | 84 | 187 |
| Caspase-6 | Molecular Function | 1 | GO:0017111 | nucleoside-triphosphatase activity | 2.345E-27 | 510 | 768 |
|  |  | 2 | GO:0016462 | pyrophosphatase activity | 7.265E-27 | 526 | 799 |
|  |  | 3 | GO:0016818 | hydrolase activity, acting on acid anhydrides, in phosphorus-containing anhydrides | 2.962E-26 | 526 | 802 |
|  |  | 4 | GO:0016817 | hydrolase activity, acting on acid anhydrides | 3.251E-26 | 527 | 804 |
|  |  | 5 | GO:0016887 | ATPase activity | 1.423E-23 | 264 | 357 |
|  |  | 6 | GO:0004386 | helicase activity | 7.077E-22 | 128 | 147 |
|  |  | 7 | GO:0030695 | GTPase regulator activity | 3.726E-21 | 322 | 464 |
|  |  | 8 | GO:0042623 | ATPase activity, coupled | 1.907E-20 | 217 | 289 |
|  |  | 9 | GO:0016772 | transferase activity, transferring phosphorus-containing groups | 4.643E-20 | 606 | 984 |
|  |  | 10 | GO:0019899 | enzyme binding | 5.429E-20 | 525 | 834 |
|  | Biological Process | 1 | GO:0006195 | purine nucleotide catabolic process | 4.882E-25 | 373 | 536 |
|  |  | 2 | GO:0022610 | biological adhesion | 1.280E-24 | 567 | 884 |
|  |  | 3 | GO:0007155 | cell adhesion | 1.280E-24 | 567 | 884 |
|  |  | 4 | GO:0072523 | purine-containing compound catabolic process | 8.714E-24 | 373 | 541 |
|  |  | 5 | GO:0030030 | cell projection organization | 1.015E-23 | 505 | 776 |
|  |  | 6 | GO:0009166 | nucleotide catabolic process | 1.181E-23 | 383 | 559 |
|  |  | 7 | GO:0009203 | ribonucleoside triphosphate catabolic process | 4.352E-22 | 338 | 487 |
|  |  | 8 | GO:0009143 | nucleoside triphosphate catabolic process | 4.503E-22 | 342 | 494 |
|  |  | 9 | GO:0009146 | purine nucleoside triphosphate catabolic process | 5.075E-22 | 339 | 489 |
|  |  | 10 | GO:0009207 | purine ribonucleoside triphosphate catabolic process | 6.657E-22 | 337 | 486 |
|  | Cellular Component | 1 | GO:0044430 | cytoskeletal part | 1.861E-25 | 689 | 1120 |
|  |  | 2 | GO:0042995 | cell projection | 6.941E-25 | 660 | 1069 |
|  |  | 3 | GO:0005730 | nucleolus | 4.021E-20 | 677 | 1133 |
|  |  | 4 | GO:0015630 | microtubule cytoskeleton | 5.935E-17 | 431 | 689 |
|  |  | 5 | GO:0043005 | neuron projection | 9.304E-17 | 373 | 583 |
|  |  | 6 | GO:0015629 | actin cytoskeleton | 1.442E-15 | 227 | 327 |
|  |  | 7 | GO:0044463 | cell projection part | 1.633E-13 | 323 | 509 |
|  |  | 8 | GO:0031012 | extracellular matrix | 6.365E-13 | 261 | 399 |
|  |  | 9 | GO:0030054 | cell junction | 6.394E-13 | 385 | 628 |
|  |  | 10 | GO:0005654 | nucleoplasm | 5.229E-12 | 748 | 1341 |
| Caspase-8 | Molecular Function | 1 | GO:0004672 | protein kinase activity | 3.651E-43 | 552 | 613 |
|  |  | 2 | GO:0016773 | phosphotransferase activity, alcohol group as acceptor | 5.211E-43 | 642 | 728 |
|  |  | 3 | GO:0016301 | kinase activity | 1.501E-36 | 727 | 854 |
|  |  | 4 | GO:0016772 | transferase activity, transferring phosphorus-containing groups | 5.082E-33 | 817 | 984 |
|  |  | 5 | GO:0004674 | protein serine/threonine kinase activity | 3.029E-28 | 387 | 432 |
|  |  | 6 | GO:0019899 | enzyme binding | 7.732E-20 | 673 | 834 |
|  |  | 7 | GO:0030695 | GTPase regulator activity | 2.978E-13 | 383 | 464 |
|  |  | 8 | GO:0004713 | protein tyrosine kinase activity | 1.204E-11 | 134 | 144 |
|  |  | 9 | GO:0060589 | nucleoside-triphosphatase regulator activity | 4.433E-11 | 387 | 477 |
|  |  | 10 | GO:0016462 | pyrophosphatase activity | 8.758E-11 | 619 | 799 |
|  | Biological Process | 1 | GO:0006468 | protein phosphorylation | 5.456E-28 | 759 | 922 |
|  |  | 2 | GO:0030030 | cell projection organization | 6.080E-20 | 631 | 776 |
|  |  | 3 | GO:0016310 | phosphorylation | 4.935E-19 | 1093 | 1422 |
|  |  | 4 | GO:0044248 | cellular catabolic process | 6.692E-17 | 1132 | 1489 |
|  |  | 5 | GO:0022610 | biological adhesion | 1.434E-16 | 700 | 884 |
|  |  | 6 | GO:0007155 | cell adhesion | 1.434E-16 | 700 | 884 |
|  |  | 7 | GO:0022008 | neurogenesis | 1.736E-16 | 794 | 1015 |
|  |  | 8 | GO:0048468 | cell development | 2.455E-16 | 956 | 1243 |
|  |  | 9 | GO:0048699 | generation of neurons | 6.089E-16 | 752 | 959 |
|  |  | 10 | GO:0031175 | neuron projection development | 2.103E-15 | 508 | 625 |
|  | Cellular Component | 1 | GO:0042995 | cell projection | 5.378E-21 | 837 | 1069 |
|  |  | 2 | GO:0015630 | microtubule cytoskeleton | 5.618E-20 | 560 | 689 |
|  |  | 3 | GO:0005730 | nucleolus | 6.422E-20 | 879 | 1133 |
|  |  | 4 | GO:0005654 | nucleoplasm | 7.907E-17 | 1014 | 1341 |
|  |  | 5 | GO:0030054 | cell junction | 1.520E-14 | 501 | 628 |
|  |  | 6 | GO:0043005 | neuron projection | 1.189E-11 | 460 | 583 |
|  |  | 7 | GO:0070161 | anchoring junction | 1.264E-10 | 165 | 187 |
|  |  | 8 | GO:0044430 | cytoskeletal part | 3.297E-10 | 832 | 1120 |
|  |  | 9 | GO:0005815 | microtubule organizing center | 4.634E-10 | 271 | 329 |
|  |  | 10 | GO:0005578 | proteinaceous extracellular matrix | 6.622E-10 | 278 | 339 |
| Granzyme B (human) | Molecular Function | 1 | GO:0016773 | phosphotransferase activity, alcohol group as acceptor | 9.593E-30 | 624 | 728 |
|  |  | 2 | GO:0016301 | kinase activity | 2.714E-29 | 719 | 854 |
|  |  | 3 | GO:0016772 | transferase activity, transferring phosphorus-containing groups | 6.301E-29 | 816 | 984 |
|  |  | 4 | GO:0004672 | protein kinase activity | 6.784E-25 | 526 | 613 |
|  |  | 5 | GO:0030695 | GTPase regulator activity | 3.570E-18 | 398 | 464 |
|  |  | 6 | GO:0060589 | nucleoside-triphosphatase regulator activity | 2.634E-16 | 404 | 477 |
|  |  | 7 | GO:0019899 | enzyme binding | 3.755E-16 | 670 | 834 |
|  |  | 8 | GO:0004674 | protein serine/threonine kinase activity | 1.568E-15 | 368 | 432 |
|  |  | 9 | GO:0008092 | cytoskeletal protein binding | 2.690E-14 | 476 | 579 |
|  |  | 10 | GO:0005083 | small GTPase regulator activity | 3.085E-14 | 269 | 307 |
|  | Biological Process | 1 | GO:0030030 | cell projection organization | 1.862E-25 | 652 | 776 |
|  |  | 2 | GO:0022008 | neurogenesis | 3.626E-21 | 819 | 1015 |
|  |  | 3 | GO:0007049 | cell cycle | 3.932E-20 | 986 | 1248 |
|  |  | 4 | GO:0044248 | cellular catabolic process | 4.306E-20 | 1160 | 1489 |
|  |  | 5 | GO:0048468 | cell development | 5.067E-20 | 982 | 1243 |
|  |  | 6 | GO:0048699 | generation of neurons | 7.085E-20 | 774 | 959 |
|  |  | 7 | GO:0031175 | neuron projection development | 2.086E-19 | 524 | 625 |
|  |  | 8 | GO:0048666 | neuron development | 3.863E-19 | 591 | 715 |
|  |  | 9 | GO:0000902 | cell morphogenesis | 4.964E-19 | 578 | 698 |
|  |  | 10 | GO:0032989 | cellular component morphogenesis | 5.106E-18 | 606 | 739 |
|  | Cellular Component | 1 | GO:0005654 | nucleoplasm | 9.447E-26 | 1059 | 1341 |
|  |  | 2 | GO:0042995 | cell projection | 2.206E-18 | 839 | 1069 |
|  |  | 3 | GO:0005730 | nucleolus | 5.721E-17 | 880 | 1133 |
|  |  | 4 | GO:0015630 | microtubule cytoskeleton | 4.771E-16 | 556 | 689 |
|  |  | 5 | GO:0045202 | synapse | 5.454E-16 | 395 | 472 |
|  |  | 6 | GO:0044451 | nucleoplasm part | 8.771E-15 | 620 | 782 |
|  |  | 7 | GO:0000267 | cell fraction | 5.326E-14 | 1101 | 1462 |
|  |  | 8 | GO:0043005 | neuron projection | 3.995E-13 | 470 | 583 |
|  |  | 9 | GO:0005626 | insoluble fraction | 3.140E-12 | 846 | 1111 |
|  |  | 10 | GO:0030054 | cell junction | 3.637E-12 | 500 | 628 |
| Granzyme B (mouse) | Molecular Function | 1 | GO:0017111 | nucleoside-triphosphatase activity | 6.942E-15 | 484 | 768 |
|  |  | 2 | GO:0016887 | ATPase activity | 1.790E-14 | 250 | 357 |
|  |  | 3 | GO:0016462 | pyrophosphatase activity | 9.010E-14 | 497 | 799 |
|  |  | 4 | GO:0030695 | GTPase regulator activity | 2.411E-13 | 309 | 464 |
|  |  | 5 | GO:0016818 | hydrolase activity, acting on acid anhydrides, in phosphorus-containing anhydrides | 2.473E-13 | 497 | 802 |
|  |  | 6 | GO:0016817 | hydrolase activity, acting on acid anhydrides | 2.615E-13 | 498 | 804 |
|  |  | 7 | GO:0016772 | transferase activity, transferring phosphorus-containing groups | 3.363E-13 | 595 | 984 |
|  |  | 8 | GO:0016301 | kinase activity | 5.339E-13 | 524 | 854 |
|  |  | 9 | GO:0060589 | nucleoside-triphosphatase regulator activity | 3.427E-12 | 313 | 477 |
|  |  | 10 | GO:0005083 | small GTPase regulator activity | 3.723E-12 | 215 | 307 |
|  | Biological Process | 1 | GO:0044282 | small molecule catabolic process | 1.164E-19 | 549 | 855 |
|  |  | 2 | GO:0044248 | cellular catabolic process | 1.976E-18 | 888 | 1489 |
|  |  | 3 | GO:0022610 | biological adhesion | 1.521E-17 | 558 | 884 |
|  |  | 4 | GO:0007155 | cell adhesion | 1.521E-17 | 558 | 884 |
|  |  | 5 | GO:0006195 | purine nucleotide catabolic process | 1.459E-16 | 360 | 536 |
|  |  | 6 | GO:0072523 | purine-containing compound catabolic process | 2.985E-16 | 362 | 541 |
|  |  | 7 | GO:0009166 | nucleotide catabolic process | 1.849E-15 | 370 | 559 |
|  |  | 8 | GO:0034656 | nucleobase, nucleoside and nucleotide catabolic process | 2.737E-15 | 378 | 574 |
|  |  | 9 | GO:0034655 | nucleobase, nucleoside, nucleotide and nucleic acid catabolic process | 2.737E-15 | 378 | 574 |
|  |  | 10 | GO:0009154 | purine ribonucleotide catabolic process | 5.049E-15 | 330 | 491 |
|  | Cellular Component | 1 | GO:0042995 | cell projection | 1.047E-13 | 635 | 1069 |
|  |  | 2 | GO:0005654 | nucleoplasm | 6.453E-13 | 774 | 1341 |
|  |  | 3 | GO:0030054 | cell junction | 2.278E-12 | 393 | 628 |
|  |  | 4 | GO:0031012 | extracellular matrix | 4.025E-12 | 265 | 399 |
|  |  | 5 | GO:0005578 | proteinaceous extracellular matrix | 7.696E-12 | 230 | 339 |
|  |  | 6 | GO:0015630 | microtubule cytoskeleton | 1.650E-11 | 423 | 689 |
|  |  | 7 | GO:0045202 | synapse | 1.603E-10 | 301 | 472 |
|  |  | 8 | GO:0044430 | cytoskeletal part | 2.295E-9 | 641 | 1120 |
|  |  | 9 | GO:0070161 | anchoring junction | 6.571E-8 | 132 | 187 |
|  |  | 10 | GO:0005912 | adherens junction | 1.379E-7 | 123 | 173 |
