# Supplementary material for: PROSPER: An Integrated Feature-Based Tool for Predicting Protease Substrate Cleavage Sites
Source: PLoS One. 2012 Nov 29;7(11):e50300. doi: 10.1371/journal.pone.0050300 (PMC3510211; doi:10.1371/journal.pone.0050300)
Supplement: Table S9 — Summary of the caspase-3 cleavage site prediction by PROSPER for huntingtin, compared with the prediction results by PoPS and SitePrediction, respectively. The top 20 ranking results of these three tools are listed, where experimentally verified cleavage sites are colored by black and bold. The cleavage score of PROSPER was generated by the regression models of PROSPER. Cleavage score of PoPS is calculated as a summation of individual scores of the P4, P3, P2, P1 and P1′ positions. The final cleavage score of SitePrediction is calculated as the product of both the frequency and similarity scores. The higher the cleavage score, the more likely a cleavage site is predicted to be cleaved. Here, “|” indicates the substrate cleavage site after the P1 position. (DOC) [file pone.0050300.s014.doc]

**Table S9**. Summary of the caspase-3 cleavage site prediction by PROSPER for huntingtin, compared with the prediction results by PoPS and SitePrediction, respectively. The top 20 ranking results of these three tools are listed, where experimentally verified cleavage sites are colored by black and bold. The cleavage score of PROSPER was generated by the regression models of PROSPER. Cleavage score of PoPS was calculated as a summation of individual scores of the P4, P3, P2, P1 and P1′ positions. The final cleavage score of SitePrediction was calculated as the product of both the frequency and similarity scores. The higher the cleavage score, the more likely a cleavage site is predicted to be cleaved. Here, “**|**” indicates the substrate cleavage site after the P1 position.

| Tool | Ranking | Cleavage position | Cleavage site (P4-P4′) | Cleavage score |
| --- | --- | --- | --- | --- |
| PROSPER | 1 | 742 | VPLD**|**TTEY | 1.00 |
|  | 2 | 2711 | VVSD\|LFTE | 0.99 |
|  | 3 | 1883 | EDSD\|LAAK | 0.92 |
|  | 4 | 2647 | EEAD\|APAP | 0.92 |
|  | **5** | **530** | **DEED\|ILSH** | **0.92** |
|  | 6 | 1881 | EEED\|SDLA | 0.91 |
|  | 7 | 456 | LEDD\|SESR | 0.90 |
|  | 8 | 144 | AESD\|VRMV | 0.90 |
|  | 9 | 1257 | VTLD\|LQNS | 0.89 |
|  | 10 | 2098 | VSPD\|KDWY | 0.89 |
|  | 11 | 545 | VPSD\|PAMD | 0.88 |
|  | 12 | 3110 | EELD\|RRAF | 0.88 |
|  | 13 | 510 | LQAD\|SVDL | 0.87 |
|  | 14 | 878 | AEID\|FRLV | 0.87 |
|  | 15 | 1388 | QEND\|TSGW | 0.86 |
|  | 16 | 2795 | LECD\|LLDD | 0.86 |
|  | 17 | 2798 | DLLD\|DTAK | 0.86 |
|  | **18** | **552** | **DLND\|GTQA** | **0.86** |
|  | **19** | **586** | **IVLD\|GTDN** | **0.85** |
|  | **20** | **513** | **DSVD\|LASC** | **0.85** |
| PoPS | **1** | **513** | **DSVD|LASC** | **16.52** |
|  | **2** | **552** | **DLND\|GTQA** | **15.71** |
|  | 3 | 878 | AEID\|FRLV | 15.34 |
|  | 4 | 1881 | EEED\|SDLA | 15.28 |
|  | 5 | 2349 | EEVD\|PNTQ | 15.27 |
|  | 6 | 456 | LEDD\|SESR | 15.07 |
|  | 7 | 2647 | EEAD\|APAP | 15.07 |
|  | **8** | **530** | **DEED\|ILSH** | **15.03** |
|  | 9 | 1628 | MHID\|SHEA | 14.47 |
|  | 10 | 2949 | AAPD\|SESV | 13.65 |
|  | 11 | 525 | SATD\|GDEE | 13.47 |
|  | 12 | 510 | LQAD\|SVDL | 12.89 |
|  | 13 | 1818 | YTLD\|SLNL | 12.88 |
|  | 14 | 1331 | SQFD\|GLSS | 12.87 |
|  | 15 | 589 | DGTD\|NQYL | 12.76 |
|  | **16** | **586** | **IVLD\|GTDN** | **12.59** |
|  | 17 | 572 | TPSD\|SSEI | 12.54 |
|  | 18 | 3089 | EQVD\|VNLF | 12.29 |
|  | 19 | 142 | CSDD\|AESD | 12.07 |
|  | 20 | 2798 | DLLD\|DTAK | 11.94 |
| SitePrediction | **1** | **513** | **DSVD|LASC** | **>99.9%** |
|  | **2** | **530** | **DEED\|ILSH** | **>99.9%** |
|  | 3 | 2647 | EEAD\|APAP | >99.9% |
|  | 4 | 2798 | DLLD\|DTAK | >99% |
|  | 5 | 1881 | EEED\|SDLA | >99% |
|  | **6** | **552** | **DLND\|GTQA** | **>99%** |
|  | 7 | 572 | EGPD\|SAVT | >99% |
|  | 8 | 2349 | EEVD\|PNTQ | >99% |
|  | 9 | 589 | DGTD\|NQYL | >99% |
|  | 10 | 525 | SATD\|GDEE | >99% |
|  | 11 | 3110 | EELD\|RRAF | >99% |
|  | 12 | 1331 | SQFD\|GLSS | >99% |
|  | **13** | **586** | **IVLD\|GTDN** | **>99%** |
|  | 14 | 456 | LEDD\|SESR | >99% |
|  | 15 | 679 | TDDD\|SAPL | >99% |
|  | 16 | 1883 | EDSD\|LAAK | >99% |
|  | 17 | 2907 | LSVD\|RVNV | >99% |
|  | 18 | 1818 | YTLD\|SLNL | >99% |
|  | 19 | 3089 | EQVD\|VNLF | >99% |
|  | 20 | 510 | LQAD\|SVDL | >99% |
